# Dynamic Shifts in ER–Plasma Membrane Junctions Signaling Define Pro‐Metastatic N‐Glycosylation and Predict Prostate Cancer Progression

**DOI:** 10.1002/advs.202522885

**Published:** 2026-02-12

**Authors:** Amanda J. Macke, Tania Kamal, Taylor E. Divita, Artem N. Pachikov, Chad A. LaGrange, Rajesh Ravichandran, Martha Morton, Robert Powers, Haowen Qiu, Jean‐Jack M. Riethoven, Colm Morrissey, Melinda Wojtkiewicz, Rebekah L. Gundry, Carol A. Casey, Armen Petrosyan

**Affiliations:** ^1^ Department of Biochemistry and Molecular Biology University of Nebraska Medical Center Omaha Nebraska USA; ^2^ The Fred and Pamela Buffett Cancer Center Omaha Nebraska USA; ^3^ Department of Pathology, Microbiology, and Immunology University of Nebraska Medical Center Omaha Nebraska USA; ^4^ Division of Urologic Surgery Department of Surgery University of Nebraska Medical Center Omaha Nebraska USA; ^5^ The Nebraska Center for Integrated Biomolecular Communication University of Nebraska‐Lincoln Lincoln Nebraska USA; ^6^ Department of Chemistry University of Nebraska‐Lincoln Lincoln Nebraska USA; ^7^ Center for Biotechnology University of Nebraska‐Lincoln Lincoln Nebraska USA; ^8^ Department of Statistics University of Nebraska‐Lincoln Lincoln Nebraska USA; ^9^ Department of Urology University of Washington Seattle Washington USA; ^10^ CardiOmics Program Center For Heart and Vascular Research and Department of Cellular and Integrative Physiology University of Nebraska Medical Center Omaha Nebraska USA; ^11^ Department of Internal Medicine University of Nebraska Medical Center Omaha Nebraska USA

**Keywords:** endoplasmic reticulum‐Plasma membrane junctions, golgi disorganization, high‐Mannose glycans, integrins, metastasis, prostate cancer

## Abstract

Prostate cancer (PCa) is the second most common and a leading cause of cancer‐related deaths among men. Current screening methods lack precision in distinguishing aggressive cases, emphasizing a need for tissue‐based biomarkers. Although Golgi disorganization, ER stress, and elevated high‐mannose (Man) glycoproteins (e.g., Integrin α_v_, key metastatic player) are recognized features of metastatic prostate tumors, their interrelationships remain unexplored. It is observed that the growth of primary prostate tumors is linked to an increase in endoplasmic reticulum (ER)–plasma membrane (PM) junctions signaling, mediated by STIM1 and ORP5. However, transition to lymph node and tissue metastasis is associated with their downregulation, loss of ER–PM communications, significant Golgi dispersal, and rapid conversion of high‐Man glycans in the Golgi to atypical MGAT5‐modified sugars that facilitate Integrin α_v_ clustering at the PM via Galectin‐3 binding. Golgi dispersal is associated with increased organelle volume and surface area to accommodate heightened trafficking and processing. These findings position STIM1 and ORP5 as biomarkers of aggressive PCa and show that high‐Man enrichment is not due to defective maturation but reflects a glycan pool that cancer cells actively utilize, suggesting that the concept of ER stress response in PCa should be redefined to include Golgi reorganization and altered ER–PM junctions.

## Graphical abstract

Prostate cancer remains a leading cause of male cancer death, yet screening cannot reliably identify aggressive disease, underscoring the need for tissue biomarkers. It is shown that primary tumors increase ER–plasma membrane junction signaling via STIM1/ORP5, whereas metastasis features their loss, Golgi dispersal, and rapid conversion of high‐mannose glycans to MGAT5‐dependent forms that promote Integrin α_v_ clustering through Galectin‐3.

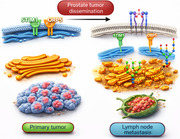

## 1 Introduction

Prostate cancer (PCa) is the second most prevalent non‐skin cancer among men in the United States [1]. Current estimates indicate approximately 268 490 new diagnoses annually and 34 500 deaths, positioning it as the second leading cause of cancer‐related mortality in men nationwide [2]. PCa is typically indolent at diagnosis, with most cases demonstrating slow progression. However, a subset (25%–30%) represents an aggressive phenotype associated with a higher likelihood of progression and poorer outcomes due to the development of metastatic castration‐resistant prostate cancer (mCRPC). This clinical variability, combined with ongoing debates about the limitations of prostate‐specific antigen (PSA) as a screening tool (particularly its inability to reliably distinguish between indolent and aggressive disease), emphasizes the urgent need for novel biomarkers [3, 4, 5]. These biomarkers would substantially improve the accuracy of detecting clinically significant cancer, potentially reducing unnecessary repeat biopsies in men with initially negative results [6]. Furthermore, understanding the aggressiveness of prostate tumors is essential for clinicians because it directly impacts treatment choices, patient care, and outcomes.

Commercially available diagnostic genetic tests, such as Confirm MDx, Prolaris, and Oncotype Dx, initially showed promise in reducing the rate of repeated prostate biopsies [7]. However, these tests are expensive and have demonstrated limitations in effectively distinguishing low‐grade PCa from more aggressive forms [8]. Moreover, positive test results do not confirm the presence of cancer with certainty and often require additional diagnostic procedures [9]. The American Urological Association advises against routinely using these tissue‐based genomic biomarkers for risk stratification or clinical decision‐making [10]. Tissue‐based diagnostic biomarkers are often more informative than blood‐ and urine‐based or imaging biomarkers because they directly reflect the tumor microenvironment and its molecular profile. One such emerging indicator is the structural integrity of the Golgi apparatus.

Golgi fragmentation is a hallmark of cancer progression. Our team identified Golgi disorganization in PCa cells and its connection to tumor advancement. We introduced the concept of the “onco‐Golgi,” where Golgi disorganization is associated with the mislocalization of Golgi resident enzymes [11]. We observed that non‐malignant prostate epithelial RWPE‐1 cells and low‐aggressive androgen‐responsive lines, LNCaP and 22Rv1, demonstrate a compact perinuclear Golgi morphology, while highly aggressive androgen‐independent, docetaxel‐resistant cells, DU145 and PC‐3, display disorganized Golgi [12, 13, 14].

Golgi scattering in advanced PCa cells leads to aberrant glycosylation and altered Golgi localization of several key proteins. First, this results in abnormal O‐glycosylation, linked to resistance to Galectin‐1‐induced apoptosis [15]. Glycogen Synthase Kinase 3 Beta (GSK3β) is also released from fragmented Golgi membranes, leading to activation of androgen receptor (AR) signaling and accelerated prostate tumor growth [16]. Furthermore, in advanced PCa cells, due to such Golgi remodeling, the proteases S1P and S2P localize to the Endoplasmic reticulum (ER) instead of the Golgi. This leads to non‐canonical activation of the Activating Transcription Factor 6 (ATF6) branch of the ER stress response, which is followed by increased proliferation [17]. Lastly, this disorganization yields altered Golgi localization of the key N‐glycosylation enzyme N‐Acetylglucosaminyltransferase III (MGAT3), promoting glycosylation of α_v_‐class integrins by MGAT3's competitor, N‐Acetylglucosaminyltransferase V (MGAT5) [12], followed by clustering of integrins and Galectin‐3 (Gal‐3) at the plasma membrane (PM) of PCa cells [18]. The former is a critical event, as it modulates tumor cell behavior, including cardinal metastatic factors, such as adhesion to extracellular matrix (ECM) and migration [19]. Despite these advances in understanding the impact of Golgi morphology on PCa, the extent to which Golgi disorganization drives the accumulation of immature glycan structures, particularly high‐mannose (high‐Man) N‐glycans, remains poorly defined.

There is growing evidence indicating that high‐Man N‐glycans are associated with tumor progression, metastasis, and poor prognosis in various cancers, including PCa (reviewed in [20]). Specifically, the elevation of high‐Man glycans is linked to prostate carcinogenesis and positively correlated with tumor grade group [21]. For instance, in mCRPC patients, expression of high‐Man glycans was upregulated [22]. However, the content of Glc1Man9, an early high‐Man oligosaccharide with a single glucose residue, whose increase indicates stalled N‐glycosylation and activation of the unfolded protein response (UPR) [23] was reduced in patients who were completely unresponsive to hormonal therapy due to neuroendocrine differentiation [22]. Additionally, recent studies reveal that high‐grade prostate tumor tissues exhibit significantly lower levels of high‐Man structures compared to Benign Prostatic Hyperplasia (BPH), low‐ and intermediate‐grade tumors [24, 25, 26]. Notably, the serum samples from primary PCa and mCRPC patients show a significant reduction in high‐Man glycans compared to those from healthy individuals [27, 28, 29].

The data from the Human Protein Atlas indicate that the expression (both at the gene and protein levels) of the key ER‐resident Man‐trimming enzyme α‐mannosidase 1A (Man1A) is not frequently downregulated in prostate adenocarcinoma [30]. In fact, many tumor samples exhibit abundant Man1A expression, indicating active trimming of high‐Man structures during conventional ER‐to‐Golgi trafficking. Additionally, rare paucimannose glycans, composed of two N‐acetylglucosamine (GlcNAc) and 1–3 Man residues, with or without a single fucose (Man(1‐3)GlcNAc2 ± Fuc), which are processed in the Golgi from high‐Man sugars, are significantly enriched in prostate tumor tissues [24]. Intriguingly, the abundance of various high‐Man glycoforms in PC‐3 cells was decreased significantly compared with LNCaP cells [31]. Together, these observations led us to hypothesize that the accumulation of high‐Man glycans in prostate tumors is not caused by defective Man cleavage due to reduced Man1A expression. Instead, we suggested that these glycans are properly processed during normal ER–Golgi–PM trafficking, an idea we then tested experimentally.

The cortical ER is a specialized region of the ER located just beneath the PM of a cell. The formation of ER–PM contact sites, or membrane contact sites (MCSs, also known as ER–PM junctions), is associated with cancer development and progression. ER–PM junctions facilitate lipid transfer between membranes, regulating Ca^2+^ influx and cell signaling [32]. Altered Ca2+ signaling has been linked to shaping PCa metastatic features and influences multiple stages of tumor progression [33]. The key structure for Ca^2+^ influx at ER–PM junctions is composed of stromal‐interacting molecule 1 (STIM1) on the ER membrane and Ca^2+^ release‐activated Ca^2+^ channel protein 1 (ORAI1) in the PM. Specifically, upon Ca^2+^ store depletion, STIM1 extends, migrates to ER–PM connections, and oligomerizes to interact with and activate ORAI1 [34]. This results in ORAI1 opening and an influx of Ca^2+^ [35].

Alterations in the formation and activity of ER–PM junctions are increasingly recognized as contributing to tumor progression. In several cancers, components of these junctions, such as STIM1 and ORAI1, are upregulated or redistributed, enhancing Ca^2^^+^ influx and lipid signaling that promote cell migration and invasion [36]. Importantly, STIM1 is overexpressed in circulating tumor cells from mCRPC tissues [37], upregulated in primary prostate tumors, and promotes PCa cell migration and invasion [38], epithelial‐to‐mesenchymal transition (EMT), and cell senescence [39]. In LNCaP cells, the loss of ORAI1 leads to increased ER‐stress‐induced apoptosis, suggesting a pro‐survival role of ER–PM junctions in prostate tumor cells [40]. However, several other observations point to alteration of ER–PM communications during cancer progression. For instance, in migrating pancreatic cancer cells, these membrane contact sites undergo regulated dissolution characterized by tether disengagement and loss of ER–PM proximity, allowing rapid spatial reprogramming of Ca^2^^+^ and lipid signaling at the leading edge [41]. STIM1 was downregulated in metastatic hepatocellular carcinoma cells [42]. STIM2 deficiency in colorectal cancer xenografts led to increased tumor size, invasion, and metastasis [43]. Next, the ER–PM junction regulator RAS association domain family member 4 (RASSF4), which promotes STIM1 recruitment and stabilization at ER–PM tethering sites [44], was found to be progressively downregulated during multiple myeloma and colon cancer progression, where its loss correlates with poor patient prognosis [45, 46].

Several families of proteins are involved in lipid exchange at the ER–PM junctions, including SMP domain proteins (extended synaptotagmins/E‐Syts and transmembrane protein 24/TMEM24), oxysterol‐binding proteins (OSBPs) and OSBP‐related proteins (ORPs), PI transfer proteins (Nir2/3), and proteins containing the glucosyltransferases, rab‐like GTPase activators, and myotubularins domain (GRAMD) [32]. Several ORPs are elevated in various cancer tissues [47], including ORP5 in metastatic lung cancer [48] and ORP8 in gastric and hepatocellular carcinomas [49]. Interestingly, ORP5 promotes cervical cancer migration and invasion by reducing ATF4‐ and ATF6‐mediated ER stress signaling [50]. While its role in PCa remains poorly characterized, data from the Human Protein Atlas indicate that ORP5 is expressed at appreciable levels in prostate tumor tissues; however, ORP8 shows low expression in prostate tumors and lacks prognostic value [51, 52]. Importantly, depletion of ORP5 may affect Golgi morphology [53], and a lack of STIM1 induces ER stress [54].

Building on this knowledge and our previous experience, we aimed to clarify the relationship between STIM1‐ and ORP5‐mediated signaling at ER–PM junctions, Golgi structure, and the expression of high‐Man glycans in primary prostate tumors and tumors that metastasized to lymph nodes (LN), visceral organs, and bones. As a reference marker, we monitored Integrin α_v_ expression. We found that as PCa progresses to high‐grade tumors, the overall level of total high‐Man glycans increases. However, the surface expression of oligomannosylated Integrin α_v_ was reduced. Transition to mCRPC was marked by the simultaneous loss of both STIM1 and ORP5, significant Golgi disorganization, and a decrease in high‐Man glycans, indicating increased processing by the standard Golgi glycosylation machinery. These findings suggest that STIM1 and ORP5 could serve as prognostic markers for PCa metastasis. They also imply that elevated high‐Man glycans do not signify defective processing but rather reflect a transitional state where an enlarged and dispersed Golgi accelerates pro‐metastatic glycosylation.

## 2 Results

### 2.1 Differential STIM1 and ORP5 Expression across Prostate Cancer Cell Lines and Tissues

**FIGURE 1:**
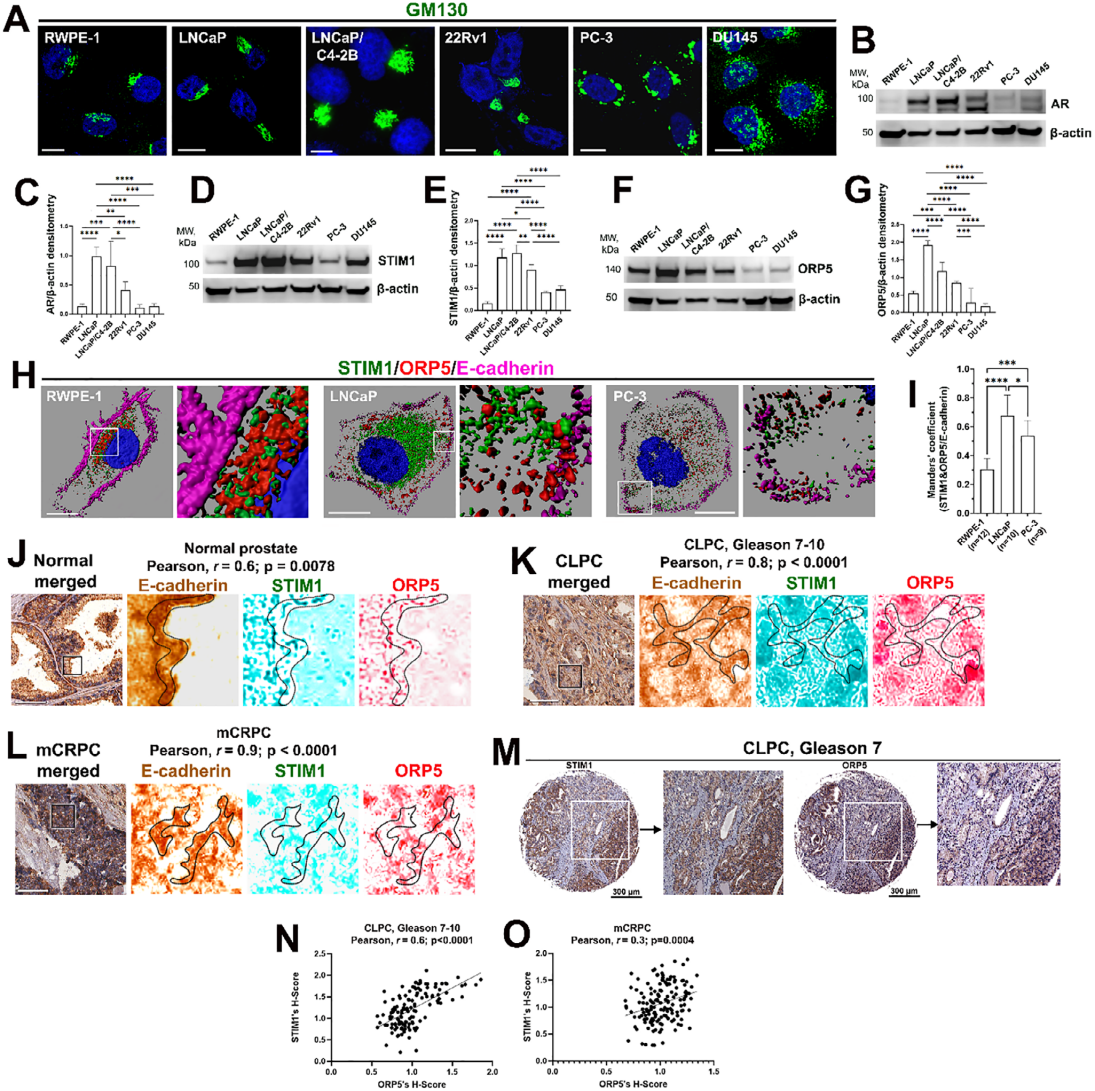
(A) Golgi (GM130) IF staining in RWPE‐1, LNCaP, LNCaP/C4‐2B, 22Rv1, PC‐3, and DU145 cells; bars, 10 µm. (B‐G) AR (B), STIM1 (D), and ORP5 (F) W‐B of lysate samples from RWPE‐1, LNCaP, LNCaP/C4‐2B, 22Rv1, PC‐3, and DU145 cells; β‐actin is a loading control. Densitometry of AR (C), STIM1 (E), and ORP5 (G) from the three independent W‐B experiments; one‐way ANOVA test. (H) Representative 3D reconstruction of STIM1 (green), ORP5 (red), and E‐cadherin (far red/magenta) SIM images of RWPE‐1, LNCaP, and PC‐3 cells; bars, 10 µm; white squares indicate an area enlarged at the right. (I) Quantification of STIM1 and ORP5 colocalization on the PM of cells in H. The STIM1 and ORP5 colocalization channel was created in Imaris. Then the colocalization of this channel with E‐cadherin was analyzed using Imaris, yielding the Manders’ overlap coefficient, which represents the amount of STIM1+ORP5 overlap colocalized with E‐cadherin; n is the number of cells counted; one‐way ANOVA test. For all statistics: ****p≤0.0001, ***p≤0.001, and *p≤0.05, mean ± SD. (J–L) Representative STIM1 (green), ORP5 (red), and E‐Cadherin (brown) IHC in normal prostate (J), PCa tissue (Gleason score 7, K), and bone metastasis sample from mCRPC patient (L); bars, 60 µm. Black boxes indicate areas magnified at right and shown as separate channels. Black lines highlight the PM regions designated by the peripheral E‐cadherin signal. Correlation analyses of PM H‐scores for STIM1 and ORP5 are displayed above each corresponding sample. (M) STIM1 and ORP5 single DAB staining in the same PCa tissue; bars, 300 µm, white squares indicate an area enlarged at the right. (N, O) Correlation analysis for STIM1 and ORP5 total H‐scores in patient‐matched PCa, Gleason 7–10 (N) or mCRPC (O) tissues.

To assess the relevance of ER–PM junctions in PCa progression, we examined STIM1 and ORP5 expression in non‐tumorigenic RWPE‐1 cells and multiple PCa lines. As previously noted, RWPE‐1 and androgen‐responsive LNCaP cells maintain a compact, juxtanuclear Golgi, whereas AR‐negative PC‐3 and DU145 cells exhibit pronounced Golgi dispersion, consistent with loss of epithelial polarity and androgen dependence [12, 13, 14]. Our current analysis extends these findings by showing that the castration‐resistant LNCaP derivative C4‐2B retains a compact Golgi (Figure 1A), aligning with their AR‐positive status and preserved polarity. Notably, C4‐2B cells express transcriptionally active AR even in the absence of ligand stimulation (Figure 1B,C) [55], positioning them as an intermediate state between androgen‐responsive and fully androgen‐independent phenotypes.

The highest expression of STIM1 and ORP5 was observed in LNCaP and C4‐2B cells. The moderate expression of both proteins was detected in another androgen‐responsive cell line, 22Rv1, while the androgen‐refractory PC‐3 and DU145 cells, which express little, if any, AR, exhibited substantially lower levels (Figure 1D–G). Consistent with these differences, upon induction of ER stress, PC‐3 and DU145 cells display impaired SOCE compared to LNCaP cells, resulting in diminished Ca^2^^+^ storage capacity and reduced Ca^2^^+^ influx [56]. Intriguingly, ORP5 is shown to act as a negative regulator of store‐operated calcium entry (SOCE) by inhibiting STIM1 clustering at ER–PM junctions through its lipid transfer activity [57]. This observation suggests a functional relationship between STIM1 and ORP5. To test this directly, we performed transient siRNA‐mediated knockdown (KD) experiments targeting STIM1 and ORP5 individually. As shown in Figure S1A,B, silencing of ORP5 or STIM1 in LNCaP cells did not significantly change the expression of the reciprocal protein. Similarly, STIM1 KD in PC‐3 and DU145 cells did not affect ORP5 levels (Figure S1D,F). However, depletion of ORP5 in these cells led to a marked increase in STIM1 expression (Figure S1C,E). Overall, these data suggest dysregulations in ER–PM junctions in highly aggressive PCa cells, given that PC‐3 and DU145 cells were derived from the distant, bone (PC‐3) [58] and brain (DU145) [59] metastatic sites, unlike the less aggressive LNCaP line, which originated from a LN metastasis.

Notably, STIM1 was originally identified as a cell‐surface protein expressed in stromal and tumor cells, where it was described as a growth regulatory molecule [60, 61]. According to the classical model, in unstimulated cells, STIM1 is uniformly distributed throughout the ER membrane and translocates to the PM only upon depletion of ER Ca^2^^+^ stores. However, emerging evidence indicates that certain cancer cells display elevated basal STIM1 expression and pre‐assembled STIM1 puncta at PM even in the absence of stimulation, implying a constitutive, “primed” state of Ca^2^^+^ signaling [56, 62]. Similarly, ORP5 was localized to ER–PM junctions in non‐stimulated (resting) cancer cells, consistent with its persistent role in lipid exchange [50, 63].

To investigate the spatial proximity of STIM1 and ORP5 at the PM of the normal prostate cells and resting PCa cells, we employed structured illumination super‐resolution microscopy (SIM) in RWPE‐1, LNCaP, and PC‐3 cells, using co‐immunostaining of STIM1 and ORP5 alongside the PM marker E‐cadherin (Figure 1H). Quantitative assessment using Manders’ overlap coefficient, which measures how much the red fluorescence signal (ORP5) overlaps with the green (STIM1) within E‐cadherin‐positive regions, showed moderate colocalization in RWPE‐1 cells. Notably, this relationship was significantly stronger in LNCaP cells, indicating increased proximity of STIM1 and ORP5 at specific PM domains. In contrast, PC‐3 cells demonstrated a marked reduction in STIM1‐ORP5 colocalization, with both proteins appearing in separate cytoplasmic puncta (Figure 1I; Movies S1 and S2), implying functional uncoupling from PM‐associated channel activity.

Next, in prostate tumor samples, we performed immunohistochemistry (IHC) with co‐staining for STIM1 and ORP5. We quantified their PM H‐scores using the widely validated Multiplex IHC v3.1.4 Cell Surface algorithm in HALO v3.4 (Indica Labs, Inc.) for quantitative membrane biomarker analysis [18, 64, 65]. To accurately assess membrane‐associated expression, E‐cadherin was used again. The E‐cadherin signal was subsequently segmented to align precisely with visually defined membrane boundaries, thereby ensuring accurate identification of cell‐surface expression patterns. In normal prostate tissue, STIM1 and ORP5 cell surface expression levels showed a moderately strong positive correlation (Pearson *r* = 0.6, p = 0.0078), indicating statistically significant co‐regulation of these two ER–PM junctional proteins under physiological conditions (Figure 1J). In both clinically localized PCa (CLPC) and metastatic samples from mCRPC patients, we detected a strong correlation between STIM1 and ORP5's PM H‐score (*r* = 0.8 and 0.9, respectively, Figure 1K,L). A similar trend was observed for their total H‐score, which was, however, lower in mCRPC samples (Figure 1M–O). Thus, despite some spatial segregation, tightly correlated expression of STIM1 and ORP5 suggests coordinated regulation of these ER–PM junction players in prostate tissues, consistent with their broader roles in maintaining cellular homeostasis.

### 2.2 Clinical Cohorts: Stage‐Dependent STIM1/ORP5 Patterns in CLPC and mCRPC

**FIGURE 2:**
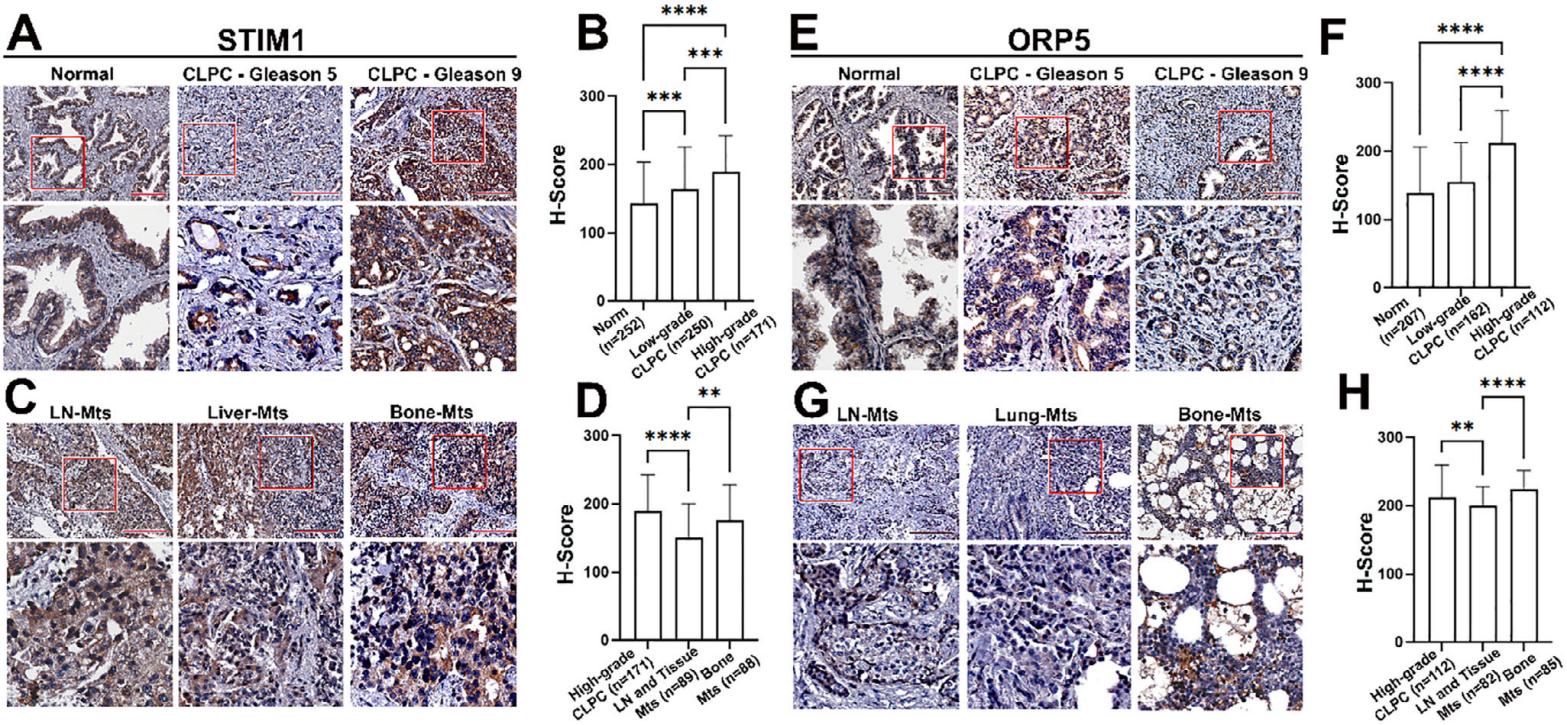
(A–D) Representative STIM1 (DAB) IHC images of tissue from (A) normal prostate of healthy donors, clinically‐localized PCa (CLPC)—Gleason 5, CLPC—Gleason 9, and (C) lymph node (LN), liver, and bone metastases from PCa patients. Quantification of the STIM1 H‐score within (B): normal prostate, low‐grade Gleason 3–7(3+4) and high‐grade Gleason 7(4+3)‐10) CLPC tumors, or (D): high‐grade CLPC, LN/soft‐tissue metastases, and bone metastases. (E‐H) Representative ORP5 (DAB) IHC images of tissue from (E) normal prostate, CLPC—Gleason 5, CLPC—Gleason 9, and (G) LN, lung, and bone metastases from PCa patients. Quantification of the ORP5 H‐score within (F): normal prostate, low‐grade and high‐grade CLPC tumors, or (H): high‐grade CLPC, LN/soft‐tissue metastases, and bone metastases. Red squares indicate an area enlarged below. IHC slides are counterstained with hematoxylin; bars, 200 µm for all images; n is the number of patients per group. For all graphs—Kruskal‐Wallis test; ****p≤0.0001, *** *p*≤0.001; ** *p*≤0.01, mean ± SD.

To further clarify the differential expression patterns of STIM1 and ORP5 in patient samples, we conducted individual DAB‐based IHC staining on CLPC and metastatic tissues. We calculated the total H‐score for both proteins, using the standard 0–300 range. We categorized CLPC patients with Gleason scores 3–6 and 7 (3+4) as low‐grade prostate tumors, while patients with Gleason scores 7 (4+3), 8, 9, and 10 were classified as high‐grade tumors. The decision to distinguish Gleason 3+4 from 4+3 was based on multiple clinical studies showing that patients with a Gleason score of 4+3 have poorer overall survival and cancer‐specific survival compared to those with a score of 3+4 [66, 67]. H‐score analysis revealed elevated STIM1 expression in both low‐grade and high‐grade primary PCa samples (Figure 2A,B). Notably, expression levels were significantly higher in high‐grade tumors compared to low‐grade counterparts. Given the similar expression profiles in LN and soft tissue metastases, these were combined into a single group. This approach is supported by genomic evidence indicating that LN and visceral metastatic lesions frequently harbor overlapping molecular signatures, which are fundamentally distinct from the molecular landscape of bone metastases [68, 69].

Compared to high‐grade CLPC, STIM1 expression was substantially lower in LN/tissue metastases but remained markedly elevated in bone metastases (Figure 2C,D). ORP5 showed a pattern of expression comparable to that of STIM1, with markedly elevated levels in high‐grade compared to low‐grade CLPC (Figure 2E,F). Similarly to STIM1, ORP5 expression declined in LN/tissue metastases relative to both high‐grade CLPC and bone metastases (Figure 2G,H). Collectively, these findings suggest that elevated STIM1 and ORP5 levels are associated with primary tumor progression. Their subsequent downregulation in LN and soft‐tissue metastases implies that disruption of ER–PM communications may accompany metastatic dissemination. Consistent with the well‐established metastatic trajectory of PCa, spreading first to regional LN and later to bone and distant soft tissues, our data indicate that STIM1 and ORP5 dynamics may reflect distinct stages of this progression cascade.

### 2.3 Fragmented Golgi Phenotype in PCa Cells Is Linked to a Lack of ER–PM Junctions

**FIGURE 3:**
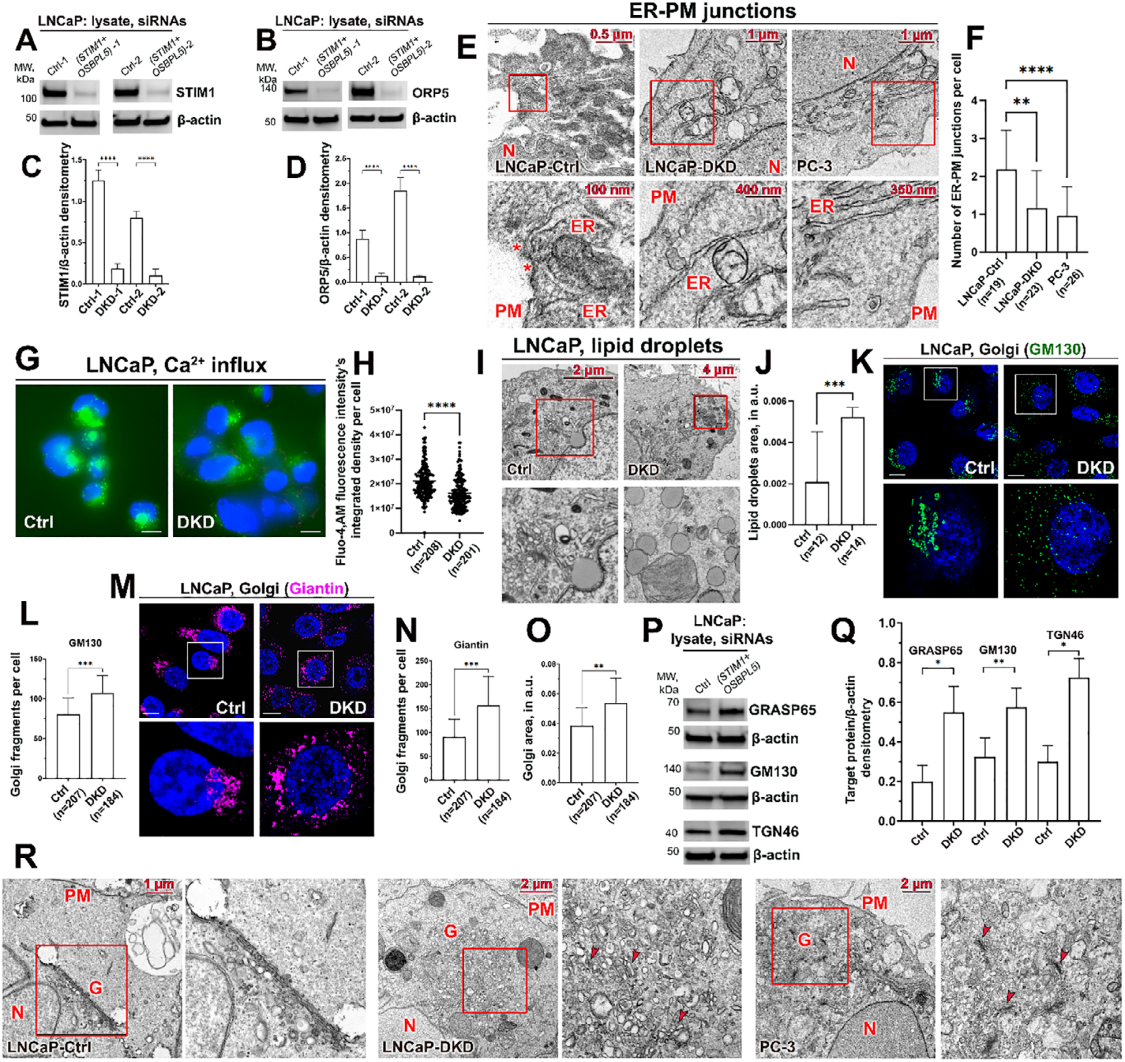
(A, B) STIM1 (A) and ORP5 (B) W‐B of lysates from LNCaP cells treated with two different sets of control or *STIM1* and *OSBPL5* siRNAs. (C, D) Densitometric quantification of STIM1 (C) or ORP5 (D) from three repeats; Welch's t test. (E) EM visualization of ER–PM junctions in LNCaP (control and DKD) and PC‐3 cells; N—nucleus, PM—plasma membrane, ER—endoplasmic reticulum. Red squares indicate an area enlarged below. Asterisks indicate ER–PM junctions. (F) Quantification of ER–PM junctions per cell from E; n—number of cells counted; Kruskal‐Wallis test, mean ± SD. (G) Representative images of the Ca^2+^ influx experiment in control and DKD LNCaP cells; bars, 10 µm. (H) Quantification of Ca^2+^‐specific Fluo‐4, AM signal from cells in G counted from three repeats; Mann‐Whitney test; median. (I) EM visualization of lipid droplets in control and DKD LNCaP cells. Red squares indicate an area enlarged below. (J) Quantification of lipid droplets in cells from I; n—number of cells counted, Welch's t test, mean ± SD. (K–N) Representative Golgi IF images of LNCaP cells treated with control or *STIM1* and *OSBPL5* siRNAs stained for GM130 (green, K) and Giantin (magenta, M); bars, 10 µm. Quantification of Golgi fragments per cell for GM130 (L) and Giantin (N) in images from K and M, respectively; Welch's t test, mean ± SD. (O) Quantification of the Golgi area per cell from M; Welch's t test, mean ± SD. (P) GRASP65, GM130, and TGN46 W‐B of lysates from LNCaP cells treated with control or *STIM1* and *OSBPL5* siRNAs. (Q) Quantification of target protein densitometry for W‐Bs represented by P; Welch's t test, mean ± SD. For all graphs: * *p*≤0.05, ** *p*≤0.01, *** *p*≤0.001, **** *p*≤0.0001. (R) EM visualization of Golgi (G) in LNCaP (control and DKD) and PC‐3 cells; N ‐ nucleus. Red squares indicate an area enlarged at the right.

Given that PC‐3 and DU145 cells, unlike LNCaP, display fragmented Golgi morphology, we investigated whether dual KD (DKD) of STIM1 and ORP5 in LNCaP cells could recapitulate the phenotype observed in these androgen‐refractory cell lines. To deplete each protein, we conducted two independent experiments using distinct siRNA mixtures (see Table S1). As shown in Figure 3A–D, these siRNA sets effectively reduced STIM1 and ORP5 expression. Subsequent experiments were conducted under both DKD conditions. Using electron microscopy (EM), we assessed ER–PM junctions. In our analysis, an actual ER–PM junction was defined as a region in which: (a) smooth ER cisterna runs parallel to the PM; (b) the intermembrane separation is ≤ 20–40 nm, consistent with well‐established morphological definitions of ER–PM contacts reported in the literature [70, 71, 72]. To ensure rigorous identification: (a) only ER profiles ≤ 40 nm from the PM were scored as junctions; (b) mitochondria‐associated membranes, vesicles, or ribosome‐rich ER not aligned with the PM were not counted.

As expected, untreated PC‐3 cells exhibited significantly fewer ER–PM contacts than control LNCaP cells. Notably, DKD in LNCaP cells also led to a marked reduction in these coupling sites, mirroring the PC‐3 phenotype (Figure 3E,F). To assess whether STIM1 depletion directly impacts PM‐mediated Ca^2^^+^ influx, we performed 30‐min time‐lapse Ca^2^^+^ imaging using the fluorescent indicator Fluo‐4 AM, following established protocols for rapid detection of intracellular Ca^2^^+^ signals [73]. This dynamic measurement confirmed a gradual increase in intracellular Ca^2^^+^ in control cells, whereas the rate of influx was significantly diminished in STIM1+ORP5DKD cells, consistent with impaired PM‐mediated Ca^2^^+^ entry (Figure 3G,H; Figure S2). In parallel, DKD cells displayed a notable accumulation of lipid droplets (Figure 3I,J), and quantification of these lipid‐rich areas confirmed a disruption in lipid homeostasis, consistent with impaired ORP5 function [74].

To assess changes in Golgi structure, we stained LNCaP cells for key Golgi matrix proteins: GM130 (*cis‐*Golgi) and Giantin (*medial*‐Golgi). In cells depleted of both STIM1 and ORP5, we observed pronounced Golgi fragmentation, evidenced by a significant increase in the number of GM130‐positive (Figure 3K,L) and Giantin‐positive (Figure 3M,N) puncta. Additionally, the total area occupied by Giantin‐positive Golgi membranes was markedly expanded (Figure 3O).

To validate this apparent increase in Golgi volume, we performed W‐B analysis to quantify GM130 expression, along with two other Golgi scaffold proteins, GRASP65 (*cis*‐Golgi) and TGN46 (*trans*‐Golgi). All three proteins showed elevated expression in DKD cells (Figure 3P,Q), supporting Golgi membrane expansion. To determine whether these increases reflected a mere expansion of Golgi mass or transcriptional activation in response to ER/Golgi stress, we also measured their mRNA levels. Notably, the transcripts of GM130, GRASP65, and TGN46 were elevated in STIM1‐ and ORP5‐depleted cells (Figure S3), suggesting that the increase in protein abundance was, at least in part, transcriptionally driven rather than solely due to membrane accumulation. This observation aligns with reports that Golgi matrix proteins are transcriptionally upregulated under ER stress conditions as part of a compensatory response to restore secretory homeostasis [75, 76, 77].

Finally, EM further confirmed these findings. Contrary to the compact, ribbon‐like Golgi observed in control LNCaP cells, DKD and PC‐3 cells exhibited not only dispersed Golgi structures but also an increased abundance of Golgi elements, indicating both disorganization and expansion of the Golgi apparatus (Figure 3R). The fragmented Golgi structures shown in panel R were identified using established morphological criteria: shortened, dispersed, and disorganized cisternae that nonetheless retained the characteristic stacked organization of the Golgi (indicated by red arrowheads).

Notably, in DU145 and PC‐3 cells, which already exhibit a scattered Golgi under basal conditions, simultaneous depletion of STIM1 and ORP5 (Figure S4A–C, H–J) further intensified this phenotype. In DU145 cells, DKD markedly increased the number of Golgi fragments per cell, as indicated by enhanced staining for GM130, Giantin, and TGN46 (Figure S4D–G). A similar effect was observed in PC‐3 cells, where DKD promoted additional Golgi dispersal, as evidenced by elevated fragmentation across the same markers (Figure S4K–N).

### 2.4 Sub‐Lethal ER Stress is Induced in Cells Lacking ER–PM Junctions

Our group has previously demonstrated that Golgi fragmentation accelerates activation of the ATF6‐mediated arm of the ER stress response [17]. The key event in this activation is the cleavage of ATF6 from the 90 kDa proform (p90) into its active 50 kDa form (p50). We examined ATF6α expression in LNCaP, PC‐3, and DU145 DKD cells and observed a substantial increase in p90 in all three cell lines (Figure S5A–C), consistent with ATF6 pathway activation in response to STIM1 and ORP5 depletion. However, the p50 cleaved fragment was elevated slightly, suggesting that ATF6 processing may still be in its early stages. Although the ATF6 axis is the only ER stress response that relies on the Golgi morphology, the other branches, PERK and IRE‐1, are of interest because of their known roles in PCa progression [78]. Notably, active IRE1‐P is elevated in all DKD cells (Figure S5D–F). Additionally, the activated PERK‐P is elevated in STIM1+ORP5 DKD LNCaP cells (Figure S5G). In contrast, PERK expression in PC‐3 and DU145 cells was barely detectable (data not shown). Interestingly, despite clear activation of ER stress pathways, none of the DKD cell lines showed signs of apoptosis. Specifically, we observed no Caspase‐3 cleavage in LNCaP, PC‐3, or DU145 DKD cells (Figure S5H–J), supporting the view that these cells are undergoing a non‐lethal, adaptive ER stress response.

To assess whether downregulation of STIM1 and ORP5 is linked to the intracellular events observed above, we next used our established model of alcohol‐induced ER stress and Golgi disorganization. Multiple international and U.S. studies link chronic alcohol use to high‐grade PCa, metastasis, and higher mortality [79, 80, 81, 82, 83, 84, 85, 86, 87], and we have previously shown that the effect of alcohol metabolites on PCa cells results in prominent Golgi dispersal [16] followed by the activation of AR signaling, ATF6 upregulation, and amplification of pro‐metastatic glycosylation [12, 17, 18]. To generate the effects of alcohol consumption in vitro, an Acetaldehyde‐generating system (AGS) was applied to the cells; this system consists of complete media supplemented with NAD^+^, ethanol (EtOH), and alcohol dehydrogenase enzyme [88]. Herein, we found that AGS‐induced Golgi fragmentation [89] is tied to reduced STIM1 and ORP5 expression in LNCaP cells (Figure S6A–E), PC‐3 (Figure S6F–H), and DU145 cells (Figure S6I,J). Thus, this alcohol‐based model of PCa progression suggests that the loss of ER–PM junctions is a critical step in the acquisition of metastatic traits by tumor cells.

### 2.5 Golgi Organization Induced by STIM1+ORP5 DKD Activates the GSK3β→HDAC6→HSP90→AR Axis

**FIGURE 4:**
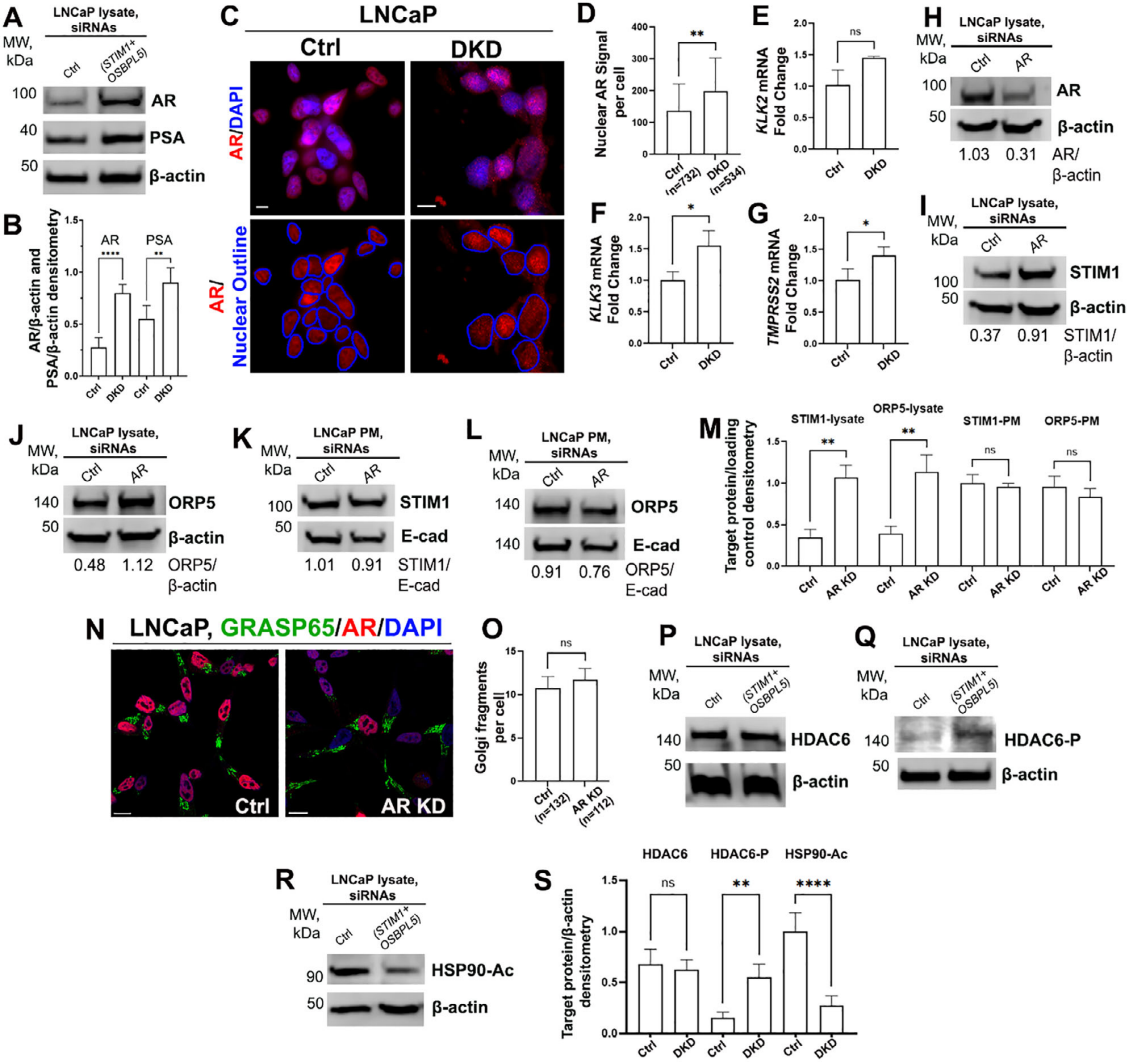
(A) AR and PSA W‐B of lysates from LNCaP cells treated with control or *STIM1* and *OSBPL5* siRNAs. (B) AR and PSA densitometry from W‐Bs represented by A; unpaired t test. (C) Representative IF images of LNCaP cells treated with control or *STIM1* and *OSBPL5* siRNAs and stained for AR (red) and nucleus (DAPI, blue). The nuclear area defined by the DAPI signal is represented by the blue outline in the lower panels; bars, 10 µm. (D) Quantification of nuclear AR signal per cell from C; n is the number of cells counted; Mann‐Whitney test. (E‐G) Quantification of fold change in mRNA expression of *KLK2* (E)*, KLK3* (F), and *TMPRSS2* (G) measured by qRT‐PCR in LNCaP cells treated with control or *STIM1* and *OSBPL5* siRNAs; Welch's t test. (H–J) AR (H), STIM1 (I), and ORP5 (J) W‐B of lysates from LNCaP cells treated with control or *AR* siRNAs. (K‐L) STIM1 (K) and ORP5 (L) W‐B of PM fraction isolated from LNCaP cells treated with control or *AR* siRNAs. (M) Quantification of densitometry for STIM1 and ORP5 in the lysates (I and J) and PM samples (K and L) from control and AR KD LNCaP samples; unpaired t test. (N) Representative IF images of control and AR KD LNCaP cells stained for GRASP65 (green) and AR (red); bars, 10 µm. (O) Quantification of the number of Golgi fragments per cell for images from N; Welch's t test. (P–R) HDAC6 (P), HDAC6‐P (Q), HSP90‐Ac (R) W‐B of lysates from LNCaP cells treated with control or *STIM1* and *OSBPL5* siRNAs. (S) Densitometry quantification for target proteins in P‐R; Welch's t test. All statistical analyses were performed using data from at least three independent experiments; * *p*≤0.05, ** *p*≤0.01, **** *p*≤0.0001, mean ± SD for all graphs.

Our previous research demonstrated that Golgi disorganization in PCa cells promotes the redistribution of GSK3β from the Golgi to the cytoplasm, leading to HDAC6 phosphorylation and preventing its nuclear import. Phosphorylated HDAC6 subsequently deacetylates HSP90, a chaperone essential for AR maturation, stability, and binding to dihydrotestosterone (DHT), thereby enhancing AR transactivation [16]. We aimed to investigate whether Golgi dispersal in STIM1 and ORP5 DKD LNCaP cells could mimic this phenomenon, considering that AR signaling is essential not only for primary prostate tumor growth but also for maintaining mCRPC progression [90]. Initial W‐B analysis showed elevated AR levels in DKD cells (Figure 4A,B), which was further supported by IF imaging revealing an increase in nuclear (active) AR following STIM1 and ORP5 depletion (Figure 4C,D). Additionally, the AR downstream protein, PSA, was highly expressed following STIM1 and ORP5 DKD (Figure 4A,B). To further understand the activation of the AR axis, we quantified mRNA expression of the AR gene targets, *KLK2*, *KLK3* (which encodes PSA), and *TMPRSS2*, by qRT‐PCR. While *KLK2* expression demonstrated no significant change (Figure 4E), both *KLK3* and *TMPRSS2* were substantially upregulated (Figure 4F,G).

Given that PC‐3 and DU145 cells lacking AR expression exhibit a fragmented Golgi phenotype, we were further interested in whether the depletion of AR in LNCaP cells can downregulate the expression of STIM1 and ORP5 and affect Golgi morphology. After siRNA‐mediated KD of AR (Figure 4H), we found instead increased expression of STIM1 (Figure 4I,M) and ORP5 (Figure 4J,M). These data prompted us to analyze whether this upregulation is associated with an increase in their content at the PM, suggesting alterations in ER–PM junctions. We isolated the PM fraction from LNCaP AR KD cells and found that the protein levels of STIM1 and ORP5 at the cell surface were similar to those in control cells (Figure 4K–M). Interestingly, cells lacking AR show no significant changes in Golgi morphology (Figure 4N,O). These findings suggest that unless the expression of STIM1 and ORP5 is compromised, the Golgi integrity is maintained in PCa cells.

We next examined AR axis activation in STIM1‐ and ORP5‐DKD cells. While total HDAC6 levels remained unchanged (Figure 4P,S), phosphorylated HDAC6 (HDAC6‐P) was significantly increased (Figure 4Q,S), and acetylated HSP90 (HSP90‐Ac) was markedly reduced (Figure 4R,S). These changes point to a role for ER–PM junction disruption in promoting AR signaling. Taken together, our findings point to an asymmetric relationship: loss of ER–PM signaling interfaces activates AR signaling, whereas AR depletion neither reduces STIM1 and ORP5 expression nor disrupts Golgi organization. Therefore, the absence of AR in PC‐3 and DU145 cells cannot be attributed to the loss of these ER–PM tethering proteins.

### 2.6 Enhanced Processing of High‐Man Glycans Leads to Abnormal MGAT5‐mediated Glycosylation

**FIGURE 5:**
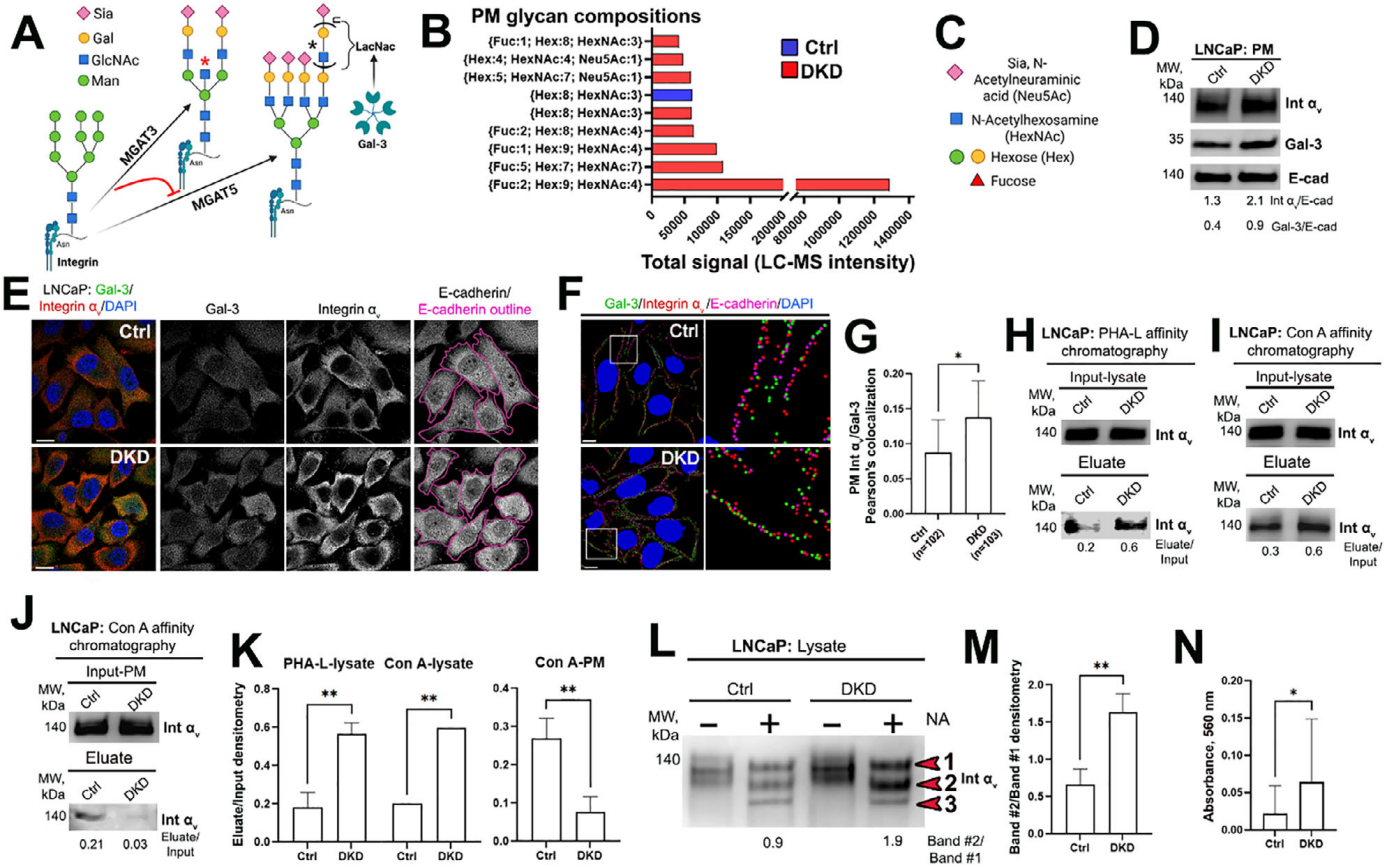
(A) Schema of conversion of high‐Man glycans in the Golgi. Bisecting GlcNAc formed by MGAT3 (red asterisk) blocks MGAT5‐mediated glycan branching. In a fragmented Golgi, loss of MGAT3 activates MGAT5. LacNAc structure generated by MGAT5 (black asterisk) has a high affinity to pentameric Gal‐3. (B) Distribution of glycan compositions in PM fractions from control and STIM1+ORP5 DKD LNCaP cells. (C) Symbol key illustrating the standardized monosaccharide representations used throughout the glycan composition plots. (D) Integrin α_v_ and Gal‐3 W‐B of PM samples from LNCaP cells treated with control or *STIM1* and *OSBPL5* siRNAs; E‐cadherin is a loading control. (E) Representative IF images of LNCaP cells treated with control or *STIM1* and *OSBPL5* siRNAs and stained for Gal‐3 (green), Integrin α_v_ (red), and E‐cadherin (magenta) as a PM marker. The magenta outline demarks the PM as highlighted for analysis; bars, 10 µm. (F) Reconstructed images of Gal‐3, Integrin α_v_, and E‐cadherin signal along the PM represented as spots; bars, 10 µm. (G) Quantification of the Pearson's *r* coefficient of colocalization for Integrin α_v_ and Gal‐3 in the highlighted PM region. Analysis was performed on the images shown in E, and represented by spots where red, green, and pink touch in F; Welch's t test. (H, I) PHA‐L (H) and Con A (I) lectins affinity chromatography of the lysate samples from control and DKD LNCaP cells. Integrin α_v_ W‐B of the input (top panel) and eluate fractions (bottom panel); input and eluate were normalized to total protein concentration. (J) Con A lectin affinity chromatography of the PM samples from control and DKD LNCaP cells. Integrin α_v_ W‐B of the input (top panel) and eluate (bottom panel); input and eluate were normalized to total protein concentration. (K) Quantification of the densitometry of the eluate/input ratio for the indicated samples from H, I, and J; unpaired t test. (L) Integrin α_v_ W‐B of the lysate samples from control and DKD LNCaP cells treated with α2‐3,6,8,9 Neuraminidase A. Arrowheads point to cleaved bands. (M) Quantification of the densitometry of the band #2/band #1 ratio; unpaired t test. (N) Serum‐starved LNCaP control and DKD cells were seeded at 1 × 10^5^ cells/well onto 48‐well plates pre‐coated with fibronectin, collagen I, collagen IV, or laminin I. After incubation, non‐adherent cells were removed, and attached cells were fixed, stained, and extracted for colorimetric quantification at OD_560_ nm. Bars represent mean optical density values averaged across all ECM substrates; paired t test. All statistical analyses were performed using data from at least three independent experiments; * *p*≤0.05, ** *p*≤0.01, mean ± SD for all graphs.

The dispersal of the Golgi following the loss of ER–PM junctions implies that another phenomenon may occur: Golgi disorganization‐mediated activation of MGAT5‐driven glycosylation [18]. Our previous study found that the increase in integrin levels on the PM of PCa cells is partially attributable to abnormal glycans, which are overrepresented due to Golgi fragmentation [18]. In particular, we found that in advanced PCa cells, loss of compact Golgi morphology results in MGAT3 localization to the ER, instead of the Golgi. This fits the literature data indicating a loss of bisecting GlcNAcylation, a specific enzymatic reaction catalyzed by MGAT3 (Figure 5A, **red asterisk**), in advanced prostate and other tumors [26, 91, 92, 93]. Meanwhile, MGAT3's alternative, MGAT5, remains in the Golgi, allowing increased MGAT5‐mediated addition of polylactosamine, containing repeats of the N‐acetyllactosamine (LacNAc) unit glycans (Figure 5A, **black asterisk**), to proteins passing through the Golgi. Specifically, in the case of α_v_ integrins, this results in their clustering at the cell surface through a tight association of LacNAc with pentameric Gal‐3 (Figure 5A). Here, we aim to determine whether the loss of ER–PM junctions decreases the delivery of high‐Man integrins to the cellular membrane and increases the proportion of integrins that undergo full MGAT5‐glycosylation in the Golgi before reaching the PM. We hypothesize that disrupting STIM1‐ and ORP5‐mediated communications between ER and PM membranes and disturbing Golgi morphology will reroute high‐Man proteins through the Golgi apparatus.

Mass spectrometry‐based glycomic analysis of PM fractions revealed a profound remodeling of N‐glycan architecture between control and DKD LNCaP cells. The control PM glycome was dominated by MGAT3‐mediated bisected complex‐type glycans, most notably (Hex:8; HexNAc:3), a canonical bisected tri‐antennary structure consistent with MGAT3 activity and a compact Golgi membrane phenotype (Figure 5B,C). In contrast, DKD cells exhibited a marked shift toward MGAT5‐modified β1,6‐branched and fucosylated complex‐type glycans, characteristic of onco‐Golgi [94, 95]. The DKD glycome was enriched in highly branched and core‐fucosylated species such as (Fuc:2; Hex:9; HexNAc:4), (Fuc:5; Hex:7; HexNAc:7), (Fuc:1; Hex:9; HexNAc:4), (Fuc:2; Hex:8; HexNAc:4), and (Hex:8; HexNAc:3), together comprising more than 90% of the total signal intensity. Among these, the tetra‐antennary, doubly fucosylated glycan (Fuc:2; Hex:9; HexNAc:4) alone contributed approximately 73% of total abundance, indicating a dominant MGAT5‐driven N‐glycan pathway (Figure 5B,C). Additionally, moderate enrichment in sialylated species (6.1%) suggested increased terminal modification by sialyltransferases, while a sharp increase in fucosylation indicates a transition to a more aggressive, castration‐resistant phenotype [96, 97]. Overall, the DKD glycome demonstrated a clear transition from bisected, MGAT3‐type glycans in control cells to hyper‐branched, fucosylated, and sialylated complex‐type glycans in DKD cells, consistent with activation of MGAT5‐ and α‐1,6‐fucosyltransferase‐mediated pathways associated with Golgi fragmentation, integrin clustering, and pro‐metastatic PM remodeling.

### 2.7 Surface Remodeling: Integrin α_v_‐Gal‐3 Clustering

Given that this glycomic shift implies enhanced MGAT5 activity and remodeling of PM architecture, we next investigated whether these changes are reflected in altered expression and spatial organization of surface glycoproteins, particularly Integrin α_v_ and Gal‐3. To validate that MGAT5‐mediated glycosylation coincides with increased integrin abundance at the PM as a result of Gal‐3‐driven clustering, we analyzed the PM fraction from DKD LNCaP cells. Indeed, W‐B analysis of this fraction revealed elevated levels of both Integrin α_v_ and Gal‐3 (Figure 5D). To confirm their association at the cell surface, we performed high‐resolution imaging of Integrin α_v_, Gal‐3, and E‐cadherin. To evaluate PM levels, the peripheral signal of E‐cadherin was used to define the outer edge of the PM in ImageJ software (represented by the PM outlines in Figure 5E), and any signal outside this line was deleted. After measuring the total signal within these lines for the whole‐cell values, the enlarge function was applied to the PM ROIs to shrink them by 10 pixels. The signal within each of these smaller ROIs was removed, leaving only the signal within the 10‐pixel‐wide ring representing the PM area. Then the signal intensity and Pearson's *r*were recorded for this PM region. The signal within the PM area was reconstructed as spots in Imaris software to illustrate the colocalization of Integrin α_v_ and Gal‐3 on the PM (Figure 5F). This confirmed an increase in association between Integrin α_v_ and Gal‐3 in cells deficient in STIM1 and ORP5 (Figure 5G), which is likely driven by MGAT5‐mediated glycosylation of integrins. Indeed, lectin chromatography of whole lysate using immobilized *Phaseolus Vulgaris Leucoagglutinin* (PHA‐L) lectin, which binds preferentially to GlcNAc residues on β1–6 branches of tri‐ or tetra‐antennary sugar chains (product of MGAT5 enzymatic reaction, Figure 5A, **black asterisk**) [98], clearly shows an elevation of integrins with this type of glycosylation (Figure 5H,K). In a similar experiment using immobilized *Concanavalin A* (Con A) lectin, which binds high‐Man glycans [99], we found that the pool of integrins carrying oligo‐Man structures is also enhanced after STIM1 and ORP5 DKD (Figure 5I,K). However, Con A chromatography of the PM fraction revealed that the subset of high‐Man integrins was markedly reduced on the PM of DKD cells (Figure 5J,K), suggesting that these integrins were converted to complex‐type glycans en route to the PM. Consistently, treatment of the lysate fraction with Neuraminidase (which removes terminal sialic acid residues) resulted in cleavage of the Integrin α_v_ band, yielding multiple lower molecular weight bands (Figure 5L, **arrowheads**). Notably, the ratio of band #2 to band #1 was significantly higher in DKD cells than in controls, indicating enhanced sialylation in the absence of STIM1 and ORP5 (Figure 5M). This finding further supports the conclusion that glycan remodeling in DKD cells predominantly follows the MGAT5 pathway, which generates more highly branched and thus more extensively sialylated structures compared to the MGAT3 pathway (Figure 5A). Consistent with this, we confirmed that such glycosylation correlates with integrin clustering, as evidenced by the enhanced adhesion of LNCaP DKD cells to ECM proteins (Figure 5N).

We further found that co‐depletion of STIM1 and ORP5 in PC‐3 cells led to a pronounced increase in Integrin α_v_‐Gal‐3 colocalization at the cell surface compared to controls (Figure S7A–C). This enhancement was also observed in DU145 cells lacking STIM1 and ORP5 (Figure S7D–F). As previously shown in androgen‐refractory PCa cells, a shift from the MGAT3 to the MGAT5 pathway occurs when MGAT3 is mislocalized due to Golgi disruption. Specifically, its translocation from the Golgi to the ER allows MGAT5 to dominate [12, 18]. To evaluate whether this mechanism operates in PC‐3 and DU145 DKD cells, we examined MGAT3 and MGAT5 expression and their subcellular localization (Figure S8A,B). In PC‐3 cells, STIM1+ORP5 depletion reduced both total MGAT3 levels (Figure S8C) and its Golgi localization (Figure S8D). In DU145 cells, although total MGAT3 expression increased (Figure S8E), its presence in the Golgi was similarly diminished (Figure S8F). In contrast, MGAT5 levels and Golgi localization remained unchanged in both cell lines following STIM1 and ORP5 DKD (Figure S8G–J). These findings indicate that loss of Golgi‐localized MGAT3 permits increased MGAT5‐mediated glycosylation, promoting Integrin α_v_‐Gal‐3 clustering at the PM and enhancing integrin retention at the cell surface.

### 2.8 Rescue of STIM1 and ORP5 Expression Restores Golgi Organization and Normalizes Integrin Glycosylation

**FIGURE 6:**
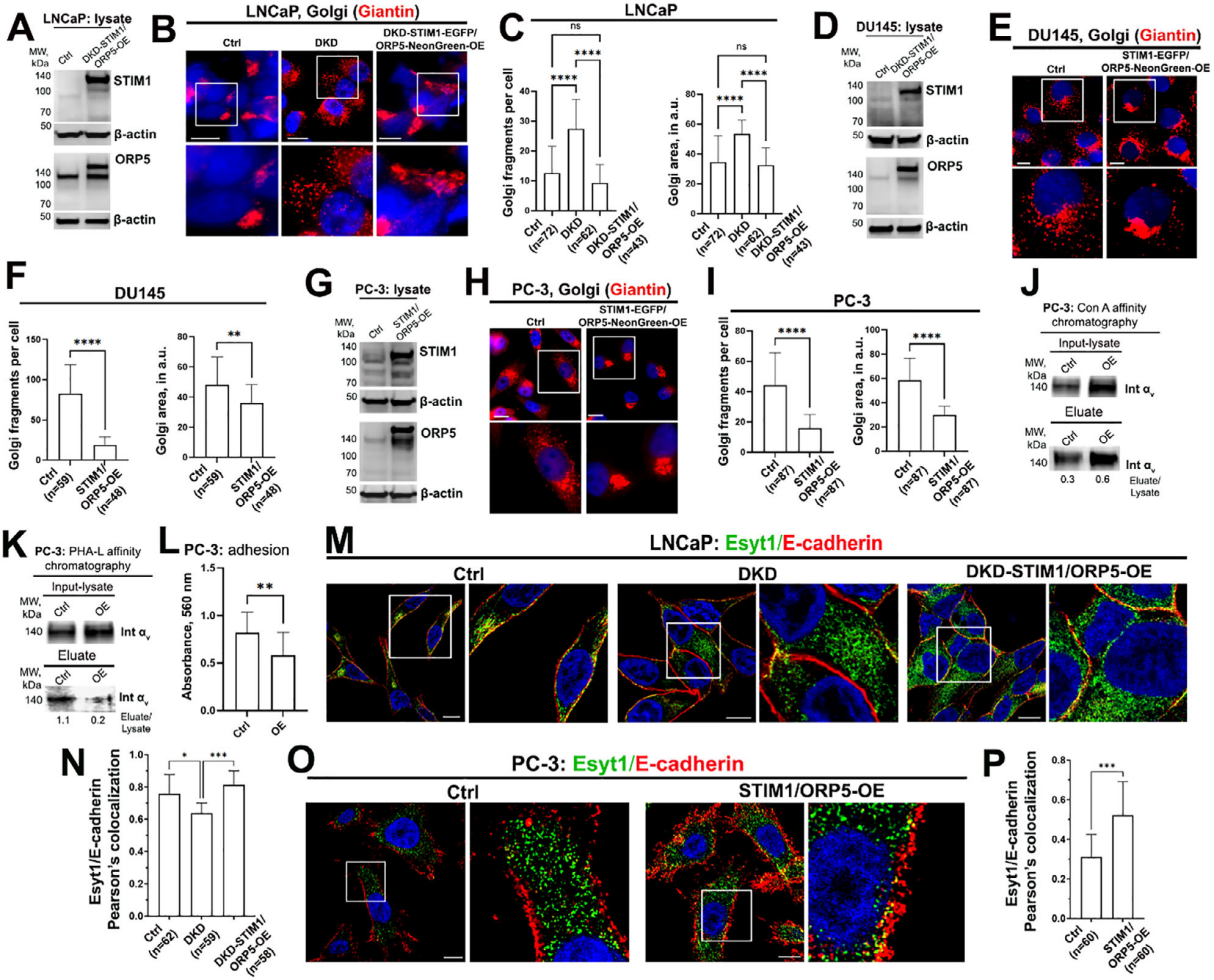
(A) STIM1 and ORP5 W‐B of the lysate from LNCaP cells overexpressed (OE) with NeonGreen‐C1‐ORP5 and STIM1‐EGFP plasmids. (B) IF staining of Golgi (Giantin) from LNCaP cells: control, DKD, and DKD followed by OE with STIM1+ORP5; bars, 10 µm. White boxes indicate the area enlarged below. (C) Quantification of Golgi fragments per cell and Golgi area per cell from B; Kruskal‐Wallis test. (D) STIM1 and ORP5 W‐B of the lysate from DU145 cells OE with NeonGreen‐C1‐ORP5 and STIM1‐EGFP. (E) IF staining of Golgi (Giantin) from DU145 cells: control and OE with STIM1+ORP5; bars, 10 µm. (F) Quantification of Golgi fragments per cell and Golgi area per cell from E; Mann‐Whitney test. (G) STIM1 and ORP5 W‐B of the lysate from PC‐3 cells OE with NeonGreen‐C1‐ORP5 and STIM1‐EGFP. (H) IF staining of Golgi (Giantin) from PC‐3 cells: control and OE with STIM1+ORP5; bars, 10 µm. (I) Quantification of Golgi fragments per cell and Golgi area per cell from H; Mann‐Whitney test. (J, K) Con A (J) and PHA‐L (K) lectins affinity chromatography of the lysate samples from control and STIM1+ORP5 OE PC‐3 cells. Integrin α_v_ W‐B of the input (top panel) and eluate fractions (bottom panel); input and eluate were normalized to total protein concentration. (L) Serum‐starved control and STIM1+ORP5 OE PC‐3 cells were seeded at 1.25 × 10^5^ cells/well onto 48‐well plates pre‐coated with fibronectin, collagen I, or collagen IV. After incubation, non‐adherent cells were removed, and attached cells were fixed, stained, and extracted for colorimetric quantification at OD_560_ nm. Bars represent mean optical density values averaged across all ECM substrates; paired t test. (M) Esyt1 and E‐cadherin IF in LNCaP cells: control, DKD, DKD followed by OE with NeonGreen‐C1‐ORP5 and STIM1‐EGFP; bars, 10 µm. (N) Quantification of colocalization between Esyt1 and E‐cadherin in cells from M. (O) Esyt1 and E‐cadherin IF in PC‐3 cells: control and OE with NeonGreen‐C1‐ORP5 and STIM1‐EGFP; bars, 10 µm. (P) Quantification of colocalization between Esyt1 and E‐cadherin in cells from O. All statistical analyses were performed using data from at least three independent experiments; * *p*≤0.05, ** *p*≤0.01, *** *p*≤0.001, **** *p*≤0.0001; mean ± SD for all graphs; n indicates number of cells.

To determine whether re‐expression of STIM1 and ORP5 can restore Golgi morphology in DKD LNCaP cells, we transfected cells (previously treated with STIM1 and ORP5 siRNAs for 72 h) with NeonGreen‐C1‐ORP5 and STIM1‐EGFP constructs. As shown in Figure 6A, expression of these exogenous proteins was markedly increased. Remarkably, overexpression of both STIM1 and ORP5 resulted in a substantial rescue of Golgi architecture: the number of Golgi fragments and the Golgi area decreased to levels comparable to those observed in control cells (Figure 6B,C).

We next examined whether overexpressing STIM1 and ORP5 could similarly reverse Golgi dispersal in DU145 and PC‐3 cells. As shown in Figure 6D–I, both cell lines overexpressing these proteins displayed a shift from a dispersed to a compact Golgi phenotype, along with a decrease in Golgi area. This effect was more pronounced in PC‐3 cells. Encouraged by these results, we next asked whether the recovered Golgi structure alters its glycosylation output. Integrins were therefore examined by lectin chromatography with Con A‐ and PHA‐L‐conjugated beads to assess changes in high‐Man and MGAT5‐modified glycans. Overexpression of STIM1 and ORP5 reduced MGAT5‐mediated β1,6‐branching, as reflected by higher Con A (high‐Man) and lower PHA‐L (complex‐type) binding (Figure 6J,K). Functional ECM adhesion assays further confirmed that these cells displayed significantly reduced adhesion (Figure 6L), consistent with decreased MGAT5‐modified integrin glycosylation. Overall, these findings show that restoring STIM1 and ORP5 expression re‐establishes Golgi integrity, normalizes integrin glycan composition, and reduces ECM adhesion functionally, confirming that ER–PM junction homeostasis is connected to Golgi organization and metastatic potential.

The EM data presented in Figure 3E,F indicate that loss of STIM1 and ORP5 in LNCaP cells reduces the number of ER–PM membrane contact sites. To validate this conclusion, we employed Esyt1, an established ER–PM contact protein, as an independent marker [100, 101]. LNCaP cells were co‐stained for Esyt1 and E‐cadherin, and cell surface‐associated Esyt1 IF was quantified following STIM1+ORP5 DKD, with or without subsequent re‐expression of these proteins. We observed a pronounced reduction in cellular membrane‐associated Esyt1 signal in STIM1+ORP5‐deficient cells, which was restored upon STIM1 and ORP5 overexpression (Figure 6M,N). Consistently, PC‐3 cells exhibited a modest cell surface Esyt1 signal that was markedly enhanced following STIM1+ORP5 overexpression (Figure 6O,P).

### 2.9 Clinical Validation of High‐Man and Integrin Associations Across Stages

**FIGURE 7:**
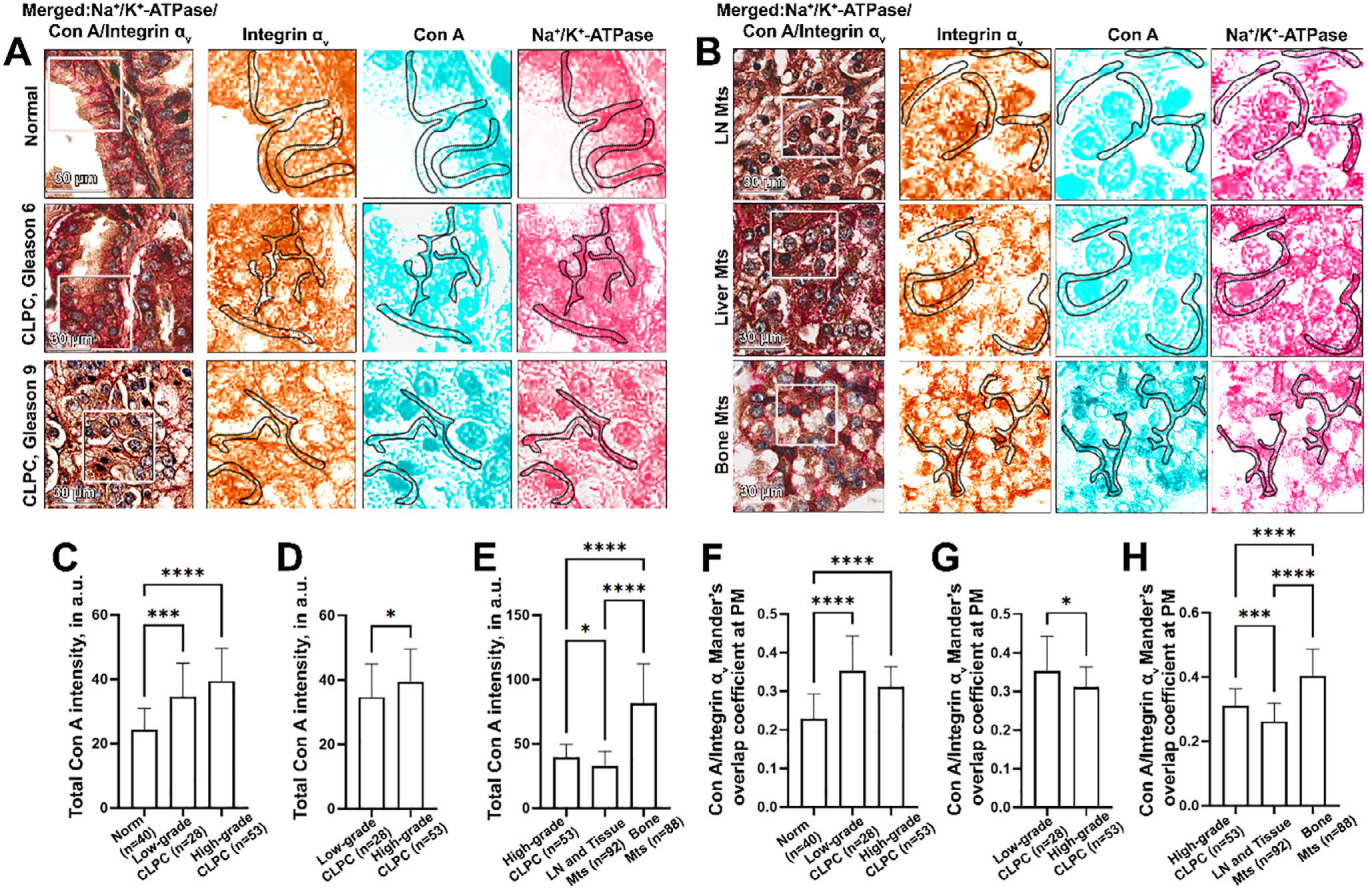
(A, B) Representative IHC images of (A) normal prostate or CLPC PCa samples and (B) LN, liver, and bone metastases samples stained for Integrin α_v_ (brown), Con A (green), and Na^+^/K^+^‐ATPase (red). White squares indicate areas that are enlarged on the right and split into three individual channels. Black lines highlight the PM regions designated by the peripheral Na+/K+‐ATPase signal; bars, 30 µm. (C–E) Quantification of total Con A intensity in the samples from A‐B; (C) multiparametric analysis between normal, low‐grade, and high‐grade CLPC, Kruskal‐Wallis test; (D) Mann‐Whitney test for low‐grade versus high‐grade CLPC; (E) multiparametric analysis between high‐grade CLPC, LN/tissue metastases, and bone metastases, Kruskal‐Wallis test. (F–H) Quantification of Integrin α_v_ and Con A Manders’ overlap coefficient on the PM in tissues from A and B: (F) multiparametric analysis between normal, low‐grade, and high‐grade CLPC, Kruskal‐Wallis test; (G) Welch's test for low‐grade versus high‐grade CLPC; (H) multiparametric analysis between high‐grade CLPC, LN/tissue metastases, and bone metastases, Kruskal‐Wallis test. For all graphs: * *p*≤0.05, *** *p*≤0.001, **** *p*≤0.0001; mean ± SD, n is the number of patients.

To corroborate these findings in a clinical context, we performed IHC co‐staining of Integrin α_v_ with Con A lectin and the PM marker, Na^+^/K^+^‐ATPase, in CLPC and mCRPC samples. We first observed that total Con A staining was significantly elevated in both low‐ and high‐grade primary prostate tumors compared to normal prostate tissue, as shown by multiparametric analysis (Figure 7A,C). Notably, high‐grade tumors exhibited a more pronounced Con A signal than low‐grade tumors (Figure 7D, Mann‐Whitney test). A pattern that emerged in mCRPC specimens mirrors the alterations seen with STIM1 and ORP5: Con A levels were decreased in LN and soft‐tissue metastases relative to high‐grade primary tumors, but markedly increased in bone metastases (Figure 7B,E).

To evaluate the association of Integrin α_v_ with Con A at the cell surface, we delineated the PM using peripheral Na^+^/K^+^‐ATPase staining, following the same segmentation approach described earlier. Manders’ overlap coefficient analysis revealed elevated Integrin α_v_‐Con A colocalization in both low‐ and high‐grade CLPC tissues compared to normal prostate (Figure 7F), paralleling the overall increase in total Con A signal. However, high‐grade tumors showed a modest but significant reduction in colocalization compared with low‐grade tumors (Figure 7G; Welch's test). This trend persisted in mCRPC samples, with reduced Integrin α_v_‐Con A overlap in LN and tissue metastases and a corresponding increase in bone metastases (Figure 7H).

Collectively, these IHC data demonstrate that although total high‐Man glycan levels are elevated in high‐grade PCa, their presence at the PM is diminished. This aligns with our previous findings showing increased MGAT5‐mediated glycosylation of integrins in advanced PCa [18]. Notably, the accumulation of total oligo‐Man structures likely reflects a buildup during the early stages of post‐translational N‐glycan processing, rather than a failure to complete glycosylation. Indeed, our results suggest that despite early retention of high‐Man forms, advanced PCa cells remain capable of completing N‐glycosylation.

An open question is whether the observed accumulation of high‐Man glycans, along with altered expression of STIM1 and ORP5 and changes in Golgi morphology, can be attributed to impaired Man trimming. To promote the formation of extended oligo‐Man glycans to varying degrees, we treated LNCaP cells with 10 µg/ml Kifunensine (Kif), a potent α‐mannosidase I inhibitor [102], for 5 days. While Kif effectively blocked α‐mannosidase activity (Figure S9A), we did not observe Golgi scattering when assessed using *cis‐* and *medial*‐Golgi markers GM130 and Giantin (Figure S9B–D). Interestingly, the Golgi area marked by these proteins was slightly but significantly enlarged (Figure S9E,F). Expression levels of STIM1 and ORP5 remained unchanged, implying that ER–PM junction integrity was not compromised (Figure S9G,H). However, ER stress sensors, ATF6 and PERK, but not IRE1, were activated in response to Kif treatment (Figure S9I,K). These findings suggest that excessive accumulation of high‐Man glycans can disrupt ER homeostasis and cause a subtle expansion of the Golgi. However, processing these sugars requires substantial disruption of Golgi architecture and ER–PM junction function.

### 2.10 Subcellular Trafficking and Redistribution of High‐Man Glycans in STIM1+ORP5‐Depleted LNCaP Cells and Prostate Tumors

**FIGURE 8:**
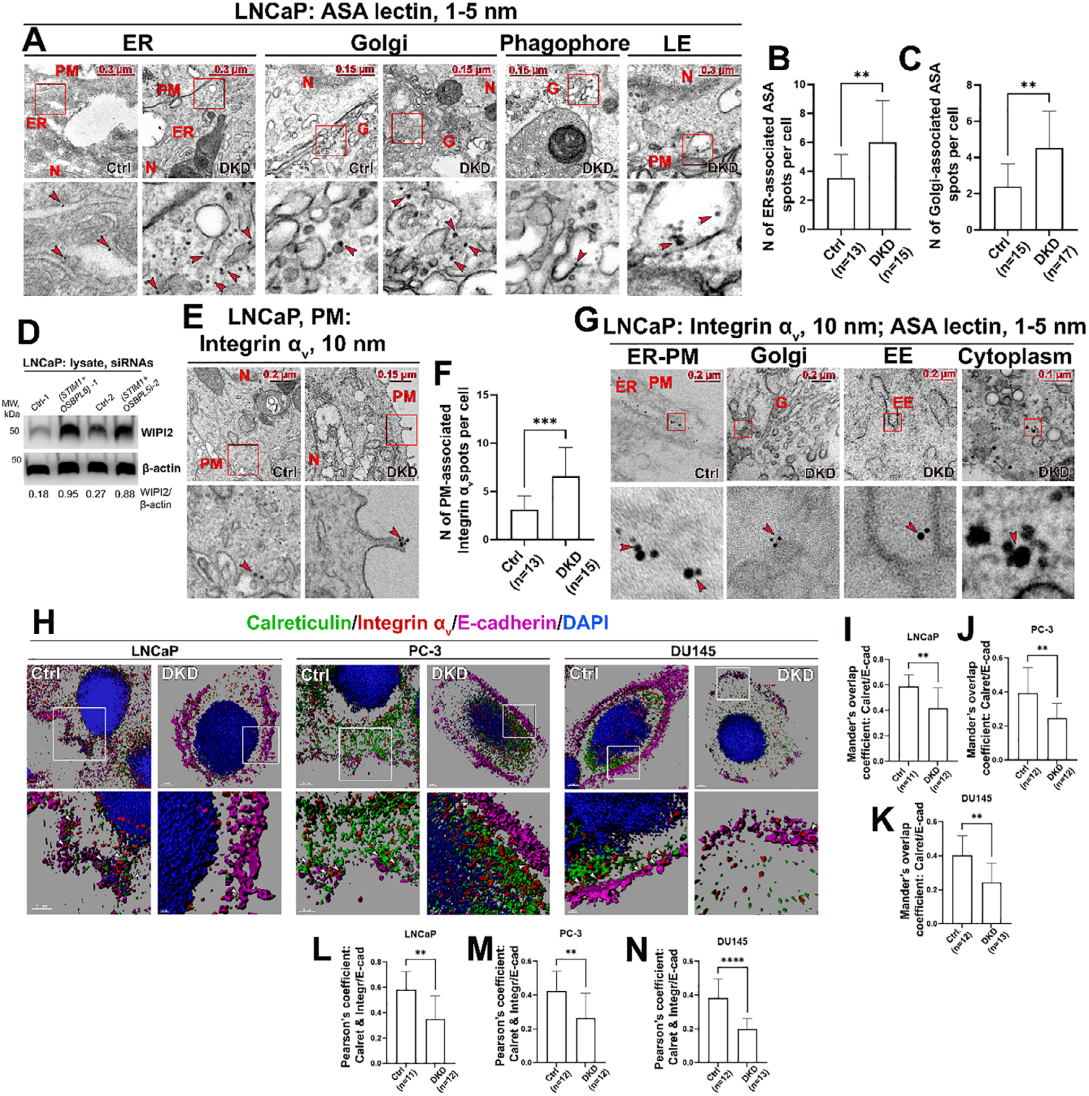
(A) EM visualization of ASA lectin in LNCaP (control and STIM1+ORP5 DKD); N—nucleus, PM—plasma membrane, ER—endoplasmic reticulum, G—Golgi, LE—late endosomes. (B, C) Quantification of the number of ER‐associated (B) or Golgi‐associated (C) ASA spots per cell, Welch's t test. (D) WIPI2 W‐B of lysate samples from control and DKD LNCaP cells treated with two different sets of *STIM1* and *OSBPL5* siRNAs. (E) EM visualization of Integrin α_v_ in control and DKD LNCaP cells. (F) Quantification of PM‐associated Integrin α_v_ spots from cells in E; Welch's t test. (G) EM visualization of Integrin α_v_ and ASA lectin merged spots in the ER–PM junctions of control LNCaP cells and Golgi, Early endosomes, and cytoplasm of DKD LNCaP cells. For all EM images: red squares indicate regions enlarged below, arrowheads highlight immunogold spots localized to the indicated organelles. (H) SIM microscopy of Calreticulin (green), Integrin α_v_ (red), and E‐cadherin (magenta) in control and DKD LNCaP, PC‐3, and DU145 cells. (I‐K) Quantification of Calreticulin colocalization with E‐cadherin in LNCaP (I), PC‐3 (J), and DU145 (K) cells; Welch's t test, n is the number of SIM images. (L‐N) Quantification of Calreticulin colocalization with Integrin α_v_ at PM in LNCaP (L), PC‐3 (M), and DU145 (N) cells; Mann‐Whitney test for LNCaP, Welch's t test for PC‐3 and DU145, n is the number of SIM images. For all graphs: ** *p*≤0.01, *** *p*≤0.001, **** *p*≤0.0001; mean ± SD.

To further characterize the subcellular distribution of high‐Man glycans in STIM1‐ and ORP5‐DKD LNCaP cells, we performed dual pre‐embedding immunogold EM. Integrin α_v_ was labeled with 10‐nm gold particles, while high‐Man glycans were detected using *Allium sativum agglutinin* (ASA) conjugated to 1–5 nm gold particles. As expected, we observed a prominent accumulation of ASA‐positive signals within the ER of DKD cells, confirming the buildup of high‐Man glycans at early stages of the secretory pathway (Figure 8A, **ER panel**; Figure 8B). Notably, ASA labeling was also elevated in the Golgi of DKD cells (Figure 8A, **Golgi panel**; Figure 8C), suggesting that these glycans continue to traffic through the conventional anterograde route despite disrupted ER–PM signaling.

We previously reported that EtOH‐induced, Golgi‐mediated autophagy (Golgiphagy) involves the recruitment of fragmented Golgi membranes as phagophore sources [103]. Consistent with this, ASA‐positive structures were absent in autophagic membranes of control LNCaP cells. However, in a subset of DKD cells, we detected ASA signals on phagophores emerging from dispersed Golgi membranes (Figure 8A, **Phagophore panel**). Golgiphagy is initiated by the WD repeat domain phosphoinositide‐interacting protein 2 (WIPI2), which promotes the conjugation of LC3 to Phosphatidylethanolamine (LC3 lipidation) by recruiting the ATG12‐5/ATG16 complex [104]. Importantly, we observed a significant upregulation of WIPI2 expression in LNCaP DKD cells with both sets of siRNAs (Figure 8D).

Additionally, numerous late endosomal (LE) structures were positive for ASA, suggesting active internalization and degradation of high‐Man glycans (Figure 8A, **LE panel**). Notably, in control LNCaP cells, Integrin α_v_ appeared as isolated gold‐labeled spots at the PM, whereas in STIM1‐ and ORP5‐DKD cells, these signals clustered prominently at the cell surface (Figure 8E,F). This aggregation supports the idea that abnormal MGAT5‐mediated glycosylation promotes Integrin clustering at the PM. Interestingly, this phenotype closely mirrors that observed in PC‐3 cells, which exhibit pronounced PM Integrin clustering in EM images [18].

Additionally, our previous study on alcohol‐induced liver injury found that high‐Man asialoglycoprotein receptor 1 (ASGPR‐1) is expressed on the PM through ER–PM communications [102]. Also, it is proposed that there are at least three distinct STIM1‐dependent components in the formation of cortical ER, which refers to the region of the ER located immediately beneath the PM: precortical structures formed within the cytosol, cortical ER closely opposed to the PM, and thin cortical ER regions lacking ribosomes [105]. In intact LNCaP cells, we identified merged Integrin α_v_ and ASA‐positive signals within both cortical and thin cortical ER–PM contact sites (Figure 8G, **ER–PM panel**). In DKD cells, merged Integrin α_v_‐ASA‐positive signals were also detected within Golgi membranes (Figure 8G, **Golgi panel**), early endosomes (Figure 8G, **EE panel**), and the cytoplasm (Figure 8G, **Cytoplasm panel**), further demonstrating the trafficking and processing of high‐Man glycans through the conventional route. Altogether, these EM findings support the concept that high‐Man sugars are not merely retained and immediately degraded in the ER, but actively participate in downstream glycosylation processes and trafficking.

One of the critical ER proteins involved in intracellular Ca^2+^ homeostasis is Calreticulin, which has a high Ca^2+^‐binding activity [106]. While it is abundantly expressed at the PM in various cell types [107], the mechanism underlying its surface localization remains poorly understood. Notably, Calreticulin expression is androgen‐regulated in prostate epithelial cells [108], but its levels are reduced in a subset of PCa, where its overexpression has been shown to suppress tumor growth and metastasis [109], highlighting its potential tumor‐suppressive function. We performed SIM imaging in LNCaP, PC‐3, and DU145 control and DKD cells to visualize the spatial co‐distribution of Integrin α_v_ and Calreticulin at the PM (Figure 8H). We observed a significant decrease in Calreticulin puncta at the PM in all DKD cell lines compared with controls, suggesting that its surface localization depends on functional ER–PM junctions (Figure 8I–K). Consistently, the colocalization between Calreticulin and Integrin α_v_ at the PM was also diminished in DKD cells (Figure 8L–N).

**FIGURE 9:**
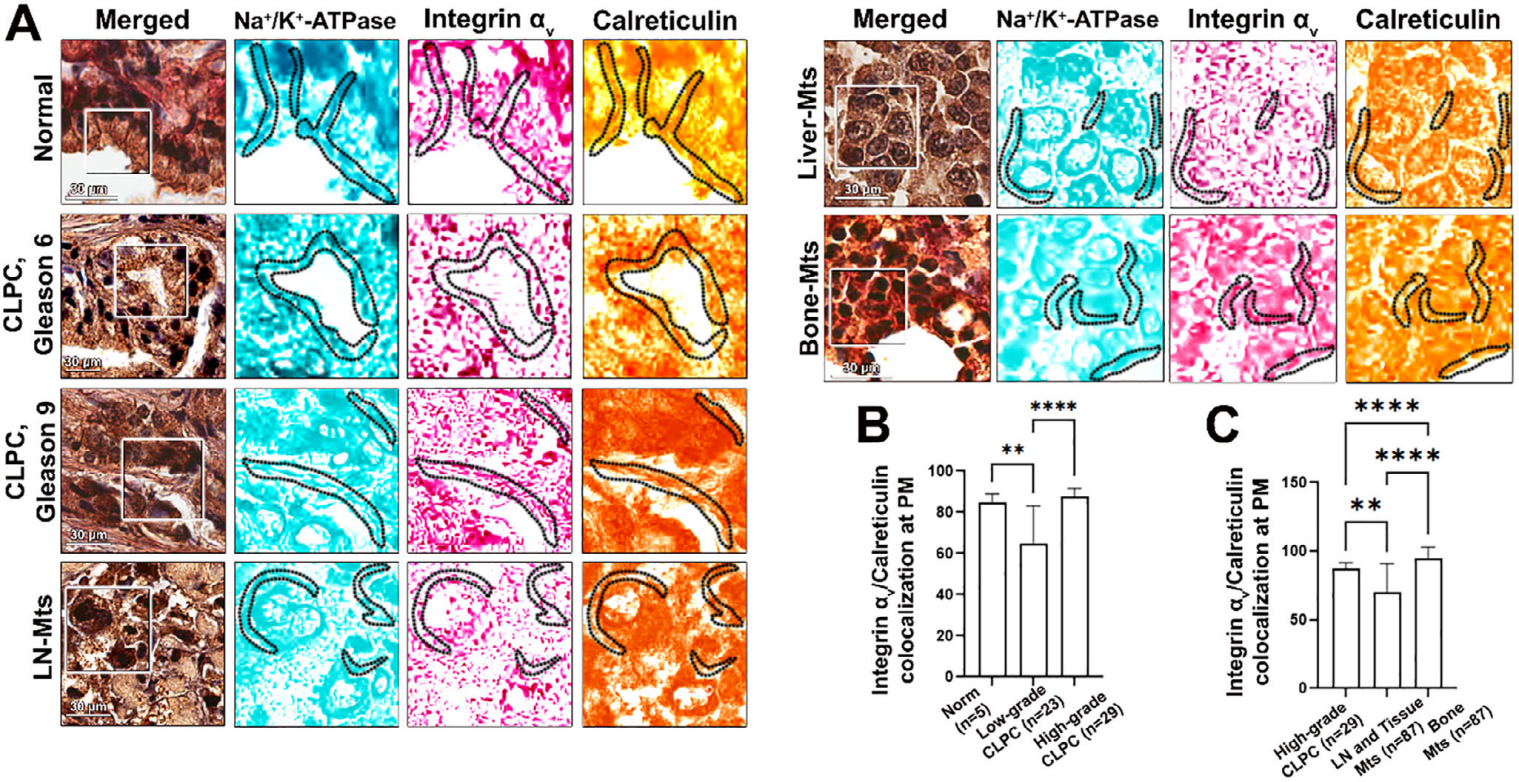
(A) Representative IHC images of normal prostate or CLPC PCa samples and lymph node (LN), liver, and bone metastases samples stained for Integrin α_v_ (red), Calreticulin (brown), and Na^+^/K^+^‐ATPase (green). White squares indicate areas that are enlarged on the right and split into three individual channels. Black lines highlight the PM regions designated by the peripheral Na+/K+‐ATPase signal. (B,C) Quantification of Integrin α_v_ and Calreticulin colocalization in the samples from A; (B) multiparametric analysis between normal, low‐grade, and high‐grade CLPC, one‐way ANOVA test; (C) multiparametric analysis between high‐grade CLPC, LN + tissue metastases, and bone metastases, Kruskal‐Wallis test. For all graphs: ** *p*≤0.01, **** *p*≤0.0001; mean ± SD, *n* is the number of patients.

To evaluate the relationship between Calreticulin and Integrin α_v_ at the PM in patient samples, we performed their IHC co‐staining along with the PM marker Na^+^/K^+^‐ATPase (Figure 9A). Interestingly, the merged Calreticulin‐Integrin α_v_ signals at the cell surface were reduced in low‐grade tumors compared to normal tissue. However, the data more closely mirrored the expression profile of STIM1/ORP5: a significant increase in high‐grade primary tumors, followed by a decrease in LN and soft tissue metastases, and then a rise in bone metastases (Figure 9B,C). Therefore, the progression to mCRPC, marked by the loss of STIM1 and ORP5, leads to the dissociation of Calreticulin‐Integrin α_v_ complexes from the PM.

### 2.11 STIM1+ORP5 Depletion Partially Drives Metabolic Reprogramming Toward mCRPC‐Like Phenotype

The conversion of androgen‐sensitive to mCRPC is accompanied by profound metabolic reprogramming that supports increased proliferation, survival, and invasiveness. Given that LNCaP cells depleted of STIM1 and ORP5 exhibit molecular and phenotypic traits reminiscent of the aggressive PC‐3 cell line, we assessed whether LNCaP DKD cells exhibit a metabolic shift toward the more aggressive PC‐3 phenotype. For this, we performed metabolite profiling of cell extracts from LNCaP control, LNCaP DKD, and PC‐3 cells (Figure S10A,B). Several metabolites showed trends suggesting that DKD cells were converging toward the PC‐3 profile. To quantify this, we conducted a directional shift analysis, where a shift estimate >0 indicates a metabolic profile closer to PC‐3, = 0 indicates equidistance between PC‐3 and control, and <0 indicates greater similarity to control.

Among the notable metabolic changes, Acetic acid levels were significantly elevated in DKD cells compared to controls, closely aligning with the PC‐3 profile (shift estimate: 0.003864; Figure S10C). Likewise, sn‐Glycero‐3‐phosphocholine was markedly reduced in both DKD and PC‐3 cells, with a shift estimate of 0.004408, further indicating metabolic convergence toward the more aggressive PC‐3 phenotype (Figure S10D). Although NAD^+^ levels were not substantially altered, the shift estimate of 0.001563 shows that DKD cells are metabolically more similar to PC‐3 than to LNCaP controls (Figure S10E). Lastly, Creatine levels in DKD cells exhibited a shift estimate of 0.02112, again pointing to greater similarity to PC‐3 (Figure S10F).

## 3 Discussion

**FIGURE 10:**
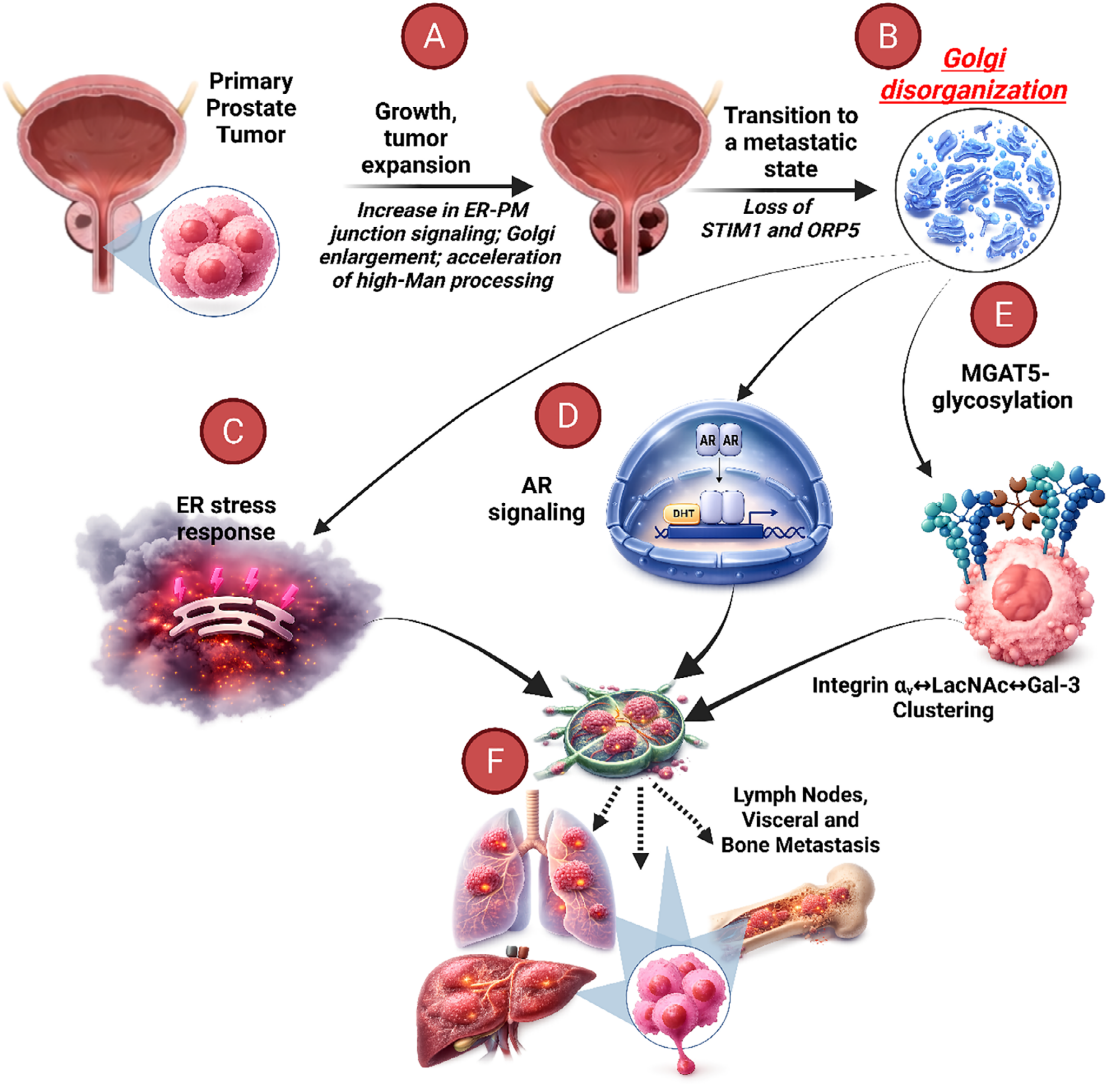
Interconnection between altered ER–PM junctions and Golgi disorganization during prostate cancer progression. (A) Primary tumor growth is characterized by increased ER–PM signaling, mediated by elevated STIM1/ORP5, and Golgi expansion, which supports enhanced protein secretion. (B) Transition to metastasis involves a reduction in STIM1/ORP5 and Golgi fragmentation, triggering: (C) ER stress response activation, particularly via the ATF6 pathway; (D) enhanced AR signaling; and (E) increased MGAT5‐mediated glycosylation, promoting Integrin‐Gal‐3 clustering at the cell surface. These events collectively enhance the metastatic potential of PCa cells (F), promoting lymph node and visceral metastases. In bone metastases, however, PCa cells often recapitulate the phenotype observed in the primary tumor.

Several important aspects of prostate tumor biology and the mechanisms underlying its critical step of metastasis are addressed in this study. First, our observation of increased STIM1 and ORP5 in high‐grade prostate tumors seems rational. Indeed, the aggressiveness of PCa cells leads to increased protein production to meet elevated metabolic demands and oxidative stress. Upregulation of ER–PM junctions could be an adaptive mechanism that restores Ca^2^^+^ homeostasis and lipid transfer, thereby promoting survival. However, excessive or oscillatory Ca^2^^+^ can activate mitochondrial permeability transition pores and caspases, leading to apoptosis of cancer cells [110]. Conversion of cancer cells to a more invasive phenotype, such as by EMT, requires the reduction of Ca^2^^+^ influx [111]. Another study substantiates that reducing ER Ca^2^^+^ store content may enable hormone‐refractory cells to evade cell death in the absence of AR stimulation [112]. These observations align with our second key finding: in LN and visceral metastases, expression of both STIM1 and ORP5 is consistently downregulated, suggesting a strategic silencing of ER–PM junction activity during metastatic progression (Figure 10).

Notably, in bone metastases, we detected upregulation of all parameters: increased STIM1, ORP5, total and PM‐specific high‐Man glycans, and Integrin α_v_ and Calreticulin association at the cell surface. Given the uniquely high extracellular Ca^2+^ concentration and signaling in the bone microenvironment [113], the sustained expression of STIM1 and ORP5 likely supports enhanced Ca^2+^ and lipid signaling, which are critical for metastatic cell survival and adaptation in this niche [114]. This environment may drive metastatic cells to maintain, or even amplify, ER–PM junction function to manage Ca^2+^ influx and membrane dynamics, which are essential for proliferation and adhesion. However, despite STIM1 and ORP5 re‐expression, our previous data showed that Integrin α_v_‐Gal‐3 colocalization was markedly reduced in bone metastases compared with LN and soft‐tissue lesions, and MGAT5‐mediated β1‐6 branching was not increased [18]. These findings indicate that, unlike earlier metastatic sites where MGAT5 activity promotes complex N‐glycan branching and Gal‐3‐dependent Integrin clustering, bone‐tropic lesions follow a distinct glycosylation program. One plausible explanation is that chronic metabolic and secretory stress within the bone niche exhausts the capacity for MGAT5‐mediated complex glycosylation, leading to the accumulation of high‐Man structures globally and at the cell surface.

Our in vitro findings provide direct evidence that alterations in ER–PM contact site architecture contribute to the phenotypes observed upon modulation of STIM1 and ORP5. While changes in STIM1 or ORP5 expression alone do not inherently define ER–PM junction structure, our combined EM and Esyt1‐based analyses demonstrate that depletion of STIM1+ORP5 leads to a measurable reduction in ER–PM contact sites, which is reversible upon re‐expression of these proteins. Notably, for the first time, we demonstrate that disruption of ER–PM communication signaling is closely associated with Golgi disorganization. This phenomenon was consistently observed in both androgen‐responsive (LNCaP) and androgen‐refractory (PC‐3 and DU145) cell lines, suggesting that functional ER–PM contact sites are essential for preserving Golgi architecture, regardless of androgen sensitivity. Our initial hypothesis was that LNCaP cells depleted of STIM1 and ORP5 would fully recapitulate the phenotype of the more aggressive, androgen‐refractory PC‐3 and DU145 cells. Indeed, DKD in LNCaP cells induced Golgi dispersal, phenocopying the fragmented Golgi architecture observed in PC‐3 and DU145 cells under steady‐state conditions. Furthermore, our glycan profiling revealed that DKD LNCaP cells undergo a shift toward MGAT5‐mediated glycosylation, leading to enhanced Gal‐3 binding and clustering of α_v_ integrins at the PM, hallmarks of a pro‐metastatic glyco‐phenotype observed in PC‐3 and DU145 cells (Figure 10). These data imply that STIM1 and ORP5 are not secondary players in the observed Golgi phenotypes but rather act as key structural determinants of ER–PM junction integrity, whose disruption propagates downstream effects on Golgi organization, Integrin glycosylation, and cell adhesion.

Surprisingly, however, this phenotypic shift was not accompanied by downregulation of AR. In contrast, DKD cells exhibited increased AR signaling. This is particularly notable given that upregulation of AR is a well‐established hallmark of mCRPC, where tumors often rewire AR signaling pathways to support survival and proliferation despite androgen deprivation. This is our third critical finding: Golgi fragmentation and loss of androgen reactivity occur as independent, parallel events during PCa progression.

Nevertheless, our findings demonstrate that STIM1+ORP5 depletion in LNCaP cells induces a metabolic reprogramming that mirrors key features of the mCRPC‐like phenotype exemplified by PC‐3 cells. The observed elevation of Acetic acid in DKD cells aligns with known increases in Acetate metabolism in advanced PCa [115], where Acetate can serve as an alternative carbon source for lipid biosynthesis and histone acetylation, thereby supporting tumor growth and epigenetic remodeling. The reduction in sn‐Glycero‐3‐phosphocholine, a choline metabolite associated with membrane lipid turnover, is also consistent with the metabolic phenotype of aggressive PCa cells, which often display altered phospholipid metabolism to meet the demands of rapid membrane synthesis and signaling [116]. Although changes in NAD^+^ levels were modest, the directional shift toward the PC‐3 profile reflects enhanced reliance on NAD^+^‐dependent processes, potentially reflecting a metabolic shift from the TCA cycle to glycolysis, which is a well‐known characteristic of mCRPC [117]. Notably, reduced creatine levels in DKD cells also trended toward a PC‐3‐like profile, consistent with previous observations that invasive PCa cells display elevated Creatine metabolism to support increased energy demands [118].

Fourth, by evaluating various intracellular stress models, we found that moderate ER stress, such as that induced by α‐mannosidase inhibition, is well tolerated by PCa cells. This level of stress did not lead to noticeable Golgi fragmentation or disruption of the ER–PM junctions’ function. In contrast, severe stress, such as that triggered by alcohol exposure, caused pronounced Golgi disorganization accompanied by downregulation of both STIM1 and ORP5. Notably, although cells lacking these two key ER–PM contact sites components exhibited activation of the ER stress response, they did not undergo apoptosis. This suggests that even in the absence of fully functional ER–PM communications, cancer cells can adapt through compensatory mechanisms.

One such mechanism is the activation of the UPR, a critical pathway that restores ER homeostasis by enhancing protein folding capacity, reducing global protein synthesis, and promoting the degradation of misfolded proteins. However, UPR alone may not fully meet the elevated biosynthetic and secretory requirements. These demands must be managed, and such adaptation requires a corresponding expansion of the Golgi, the central hub for protein processing and trafficking [119]. Importantly, such expansion cannot occur in a compact Golgi ribbon structure and is only achievable when the Golgi is dispersed, allowing increased surface area and spatial flexibility to accommodate heightened trafficking and processing loads [120]. The supply of new Golgi membranes is tightly regulated, in part, by steady‐state Golgiphagy, which prevents excessive accumulation and maintains Golgi homeostasis. Therefore, we propose revisiting the classical concept of the UPR to include this critical structural and functional reorganization of the Golgi as an integral component of the cellular stress response.

Fifth, in cells that lack ER–PM junctions, Golgi dispersal appears to be a key adaptive response that helps them cope with aggregated glycoproteins accumulating in the ER. In this setting, a dispersed Golgi can accelerate MGAT5‐dependent N‐glycosylation, in part because MGAT3 is lost. While this shift promotes abnormal integrin glycosylation and pro‐metastatic signaling, it may simply be the price cells pay to avoid otherwise lethal ER stress. Importantly, despite an overall increase in high‐Man glycans, we observed reduced cell‐surface expression of these glycans, together with increased global sialylation, consistent with enhanced MGAT5‐driven complex glycosylation. Collectively, these data suggest that the elevated high‐Man signatures reported in many cancers may primarily reflect transitory ER accumulation of glycoproteins rather than defective Golgi maturation. Nevertheless, we cannot exclude the possibility that a portion of high‐Man‐carrying glycoproteins escapes conventional processing and reaches the PM via non‐canonical trafficking pathways, as supported by our observation of the Integrin α_v_’s PM fraction sensitivity to Endo‐H digestion (data not shown). However, the detection of high‐Man glycans and Integrin α_v_‐ASA merged spots within early and late endosomal compartments strongly suggests that they are subject to lysosomal degradation. Additionally, degradation via macroautophagy, including Golgiphagy, remains a plausible complementary route for the clearance of these unprocessed glycoproteins.

Another intriguing aspect of our findings on high‐Man glycan turnover is the accumulation of these glycan species at ER–PM junctions in cortical regions. Observations in HEK293 cells show that vesicular stomatitis virus G‐protein (VSVG) is delivered to the cell surface at these sites immediately after Golgi exit, revealing that ER–PM contact zones serve as trafficking hubs for membrane protein transport to and from the PM [121]. In this context, we observed a close association between high‐Man‐bearing integrins and Calreticulin within these structures. This observation aligns with multiple lines of evidence, including those from PCa cells, indicating that the interaction between Calreticulin, a key ER‐resident chaperone, and various Integrin family members is essential for Integrin stability [122, 123, 124]. Here, we detected that in cells depleted from STIM1 and ORP5, Calreticulin was segregated from PM, indicating that highly likely the role of this molecular chaperone in ER–PM junctions is to serve as a quality control or retention checkpoint: the cortical regions of ER–PM contact domains may act as pre‐Golgi quality control stations where high‐Man integrins are temporarily retained before further processing. Although the possibility exists that these integrins may be exposed to the cell surface via ER–PM contacts, we did not observe any ultrastructural (EM) evidence supporting this route of delivery. Given the known tight association between integrins and the cytoskeleton [125], it would be interesting to investigate in the future whether underglycosylated integrins contribute to the stabilization or formation of ER–PM junctions. In our preliminary experiments, depletion of Integrin α_v_ did not reduce STIM1 or ORP5 levels, but we cannot rule out a regulatory relationship. At present, we can only speculate that the role of ER–PM signaling interfaces in glycoprotein dynamics extends beyond serving as a passive reservoir for high‐Man‐bearing proteins.

## 4 Materials and Methods

### 4.1 Patient Tissue Samples, Ethics Approval, and Consent to Participate

The tissue sections from normal prostate and PCa patients were obtained from Tissue Array (TissueArray.Com LLC) and Novus Biological. Also, sections were provided through the Department of Pathology and Microbiology (IRB protocol # 304‐16‐EP) at the University of Nebraska Medical Center, and metastatic tumor samples were obtained from patients who died from CRPC under the aegis of the Prostate Cancer Donor Program at the University of Washington (IRB protocol #2341). All procedures performed in studies involving human participants were in accordance with the ethical standards of the institutional and/or national research committee and with the 1964 Helsinki declaration and its later amendments or comparable ethical standards. Informed consent was obtained from all individual participants included in the study.

### 4.2 Cell Culture

The androgen‐responsive prostate epithelial cell line, RWPE‐1 (RRID:CVCL_3791), was used to represent normal prostate epithelium. The LNCaP (RRID:CVCL_1379) and 22Rv1 (RRID:CVCL_1045) cell lines are androgen‐responsive prostate cancer cells that exhibit a compact perinuclear Golgi. The PC‐3 (RRID:CVCL_0035) and DU145 (RRID:CVCL_0105) cell lines are aggressive, androgen‐refractory lines characterized by a disorganized Golgi. All cell lines were authenticated and regularly tested to ensure they were free of mycoplasma and other contaminants. Therefore, inclusion of these cell lines is essential to support the study's conclusions on the relationship between Golgi structure and metastatic potential.

### 4.3 Quantification and Statistical Analysis

Statistical analyses were performed using Microsoft Excel, GraphPad Prism 9.0 (GraphPad Software), and R software. Prior to analysis, the raw datasets were examined for quality control, including the evaluation of potential outliers and the assessment of distributional assumptions when applicable. Data were presented as mean ± SD, unless otherwise indicated (e.g., median‐based analyses for nonparametric comparisons). The sample size (n), definition of n (biological replicates, cells, images, or patients), and the statistical test applied are specified in the corresponding figure panels and figure legends. Multi‐parametric analyses performed in R included: a) Tukey's multiple comparisons test with Benjamini–Hochberg false discovery rate (FDR) adjustment; b) Kruskal–Wallis testing followed by Dunn's post hoc multiple comparisons, with Benjamini–Hochberg adjustment; c) pairwise Wilcoxon rank‐sum exact tests, with Benjamini–Hochberg adjustment; and d) pairwise t‐tests, with Benjamini–Hochberg adjustment. Additional analyses performed in Prism included Mann–Whitney tests, unpaired two‐sided Student's *t*‐tests, and one‐way or two‐way ANOVA, with post hoc testing as indicated in the figure legends. Unless otherwise specified, all statistical tests were two‐sided, and statistical significance was defined as *p*≤0.05. Exact *p*‐values were reported in the figures and figure legends.

The remaining Materials and Methods are detailed in the Supplemental Materials and Methods section and include the following: a) Antibodies and Reagents; b) siRNA transfection; c) Fluo‐4, AM Live Calcium Staining; d) RNA Isolation; e) One‐Step Quantitative Real‐Time Polymerase Chain Reaction (qRT‐PCR); f) Determination of α‐Mannosidase Activity; g) Plasma Membrane Protein Isolation; h) Lectin‐Affinity Isolation; i) Endoglycosidase H Digestion; j) Neuraminidase Digestion; k) Metabolomic Analysis; l) Immunofluorescent Staining; m) Immunohistochemistry (IHC); n) Immunogold Electron Microscopy; o) Mass Spectrometry N‐Glycan Analysis; p) Plasmid DNA Extraction and Transfection; q) Adhesion Assay.

## Funding

A.P. was supported by NIH grant NIAAA R01AA027242 and Fred and Pamela Buffett Cancer Center pilot project; R.P. and J.M.R. were supported by NIGMS 1P20GM113126. C.A.C. was funded by VA Merit Award (I01BX004171, NIAAA R01AA020735, and F31AA031186) VA Research Career Scientist Award (IK6BX004853; R.L.G. was supported by NIH R35‐HL155460).

## Conflicts of Interest

The authors declare no conflicts of interest.

## Supporting information




**Supporting File 1**: advs74262‐sup‐0001‐SuppMat.docx.

**Supporting File 2**: advs74262‐sup‐0002‐MovieS1.mov.

**Supporting File 3**: advs74262‐sup‐0003‐MovieS2.mov.

## Data Availability

The datasets used and/or analyzed during the current study are available from the corresponding author upon reasonable request.
