# Supplementary material for: Dynamic Shifts in ER–Plasma Membrane Junctions Signaling Define Pro‐Metastatic N‐Glycosylation and Predict Prostate Cancer Progression
Source: Adv Sci (Weinh). 2026 Feb 12;13(30):e22885. doi: 10.1002/advs.202522885 (PMC13248819; doi:10.1002/advs.202522885)
Supplement: Supplementary file 1 — Supporting File 1: advs74262‐sup‐0001‐SuppMat.docx. [file ADVS-13-e22885-s002.docx]

**Dynamic Shifts in ER–Plasma Membrane Junctions Signaling Define pro-metastatic N-Glycosylation and Predict Prostate Cancer Progression**

Amanda J. Macke^1,2^, Tania Kamal^1,2^, Taylor E. Divita^1,2^, Artem N. Pachikov^3^, Chad A. LaGrange^4^, Rajesh Ravichandran^5,6^, Martha Morton^5,6^, Robert Powers^5,6^, Haowen Qiu^7^, Jean-Jack M. Riethoven^7,8^, Colm Morrissey^9^, Melinda Wojtkiewicz^10^, Rebekah L. Gundry^10^, Carol A. Casey^11^, and Armen Petrosyan^1,2,*^

Supporting Information

Supplemental Figures


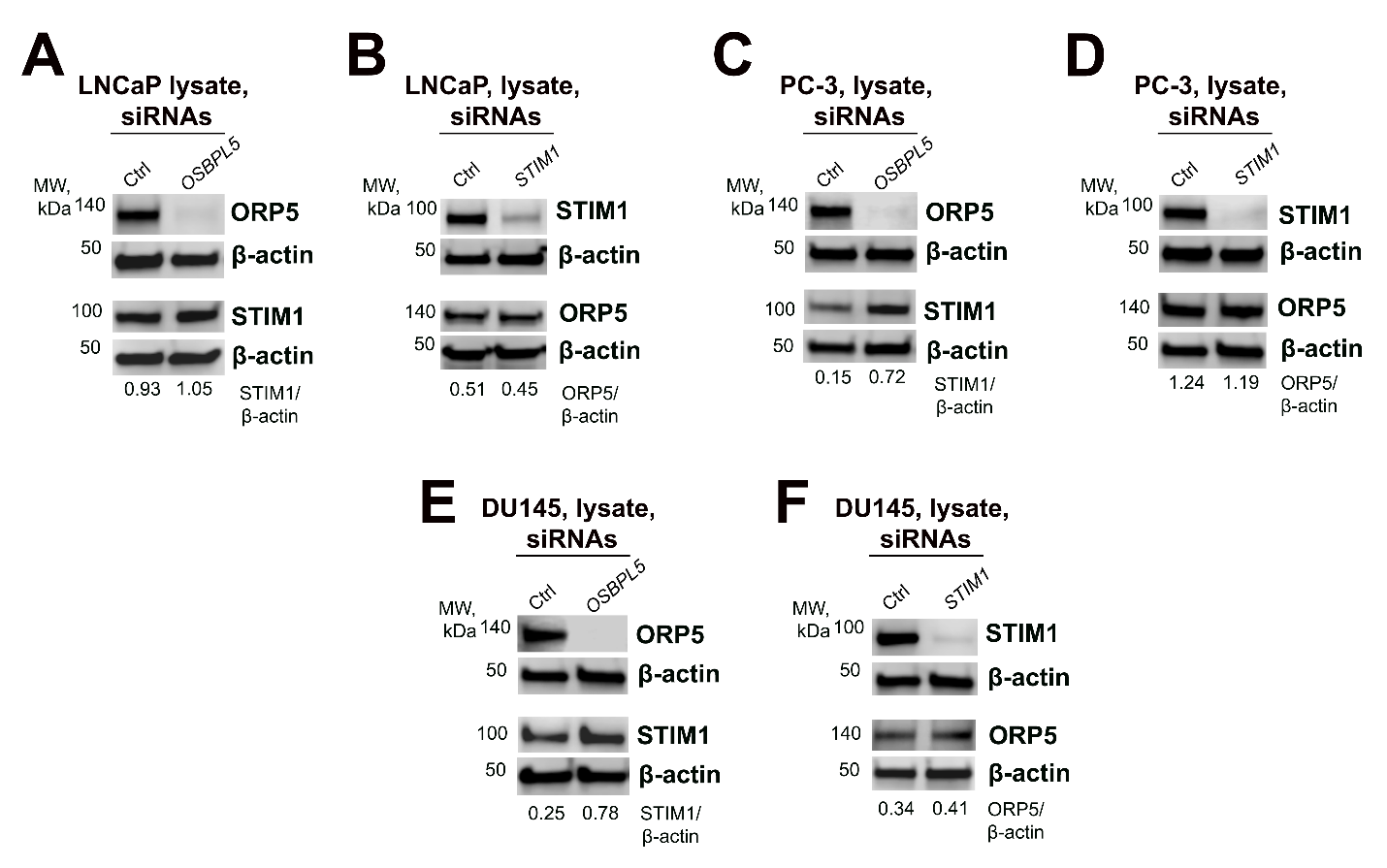


**Figure S1**. **(A)** STIM1 and ORP5 W-B of lysates from LNCaP cells treated with Ctrl or *OSBPL5* siRNAs. **(B)** STIM1 and ORP5 W-B of lysates from LNCaP cells treated with Ctrl or *STIM1* siRNAs. (**C)** STIM1 and ORP5 W-B of lysates from PC-3 cells treated with Ctrl or *OSBPL5* siRNAs. **(D)** STIM1 and ORP5 W-B of lysates from PC-3 cells treated with Ctrl or *STIM1* siRNAs. **(E)** STIM1 and ORP5 W-B of lysates from DU145 cells treated with Ctrl or *OSBPL5* siRNAs. **(F)** STIM1 and ORP5 W-B of lysates from DU145 cells treated with Ctrl or *STIM1* siRNAs.


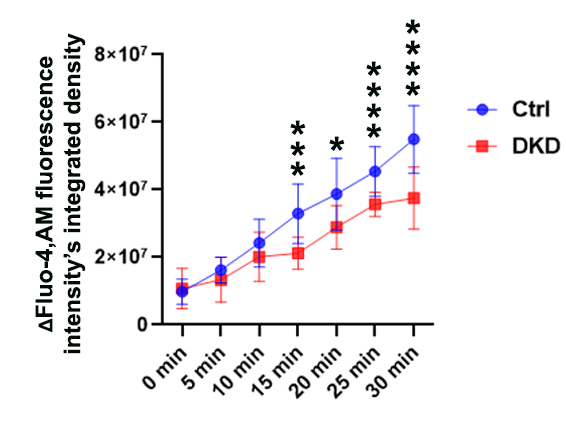


**Figure S2.** **Normalized Fluo-4 time-course confirms impaired Ca²⁺ influx in DKD cells.** Fluo-4 fluorescence was monitored every 5 min for 30 min. For each condition, 0-min fluorescence was used as baseline (F₀), and all values were normalized as ΔF = F(t) − F₀. Control cells show a continuous elevation of ΔF, while DKD cells display a markedly reduced rise, consistent with disrupted PM Ca²⁺ influx. Mean ± SD; statistical significance determined by multiple t test; * p≤0.05, *** p≤0.001, **** p≤0.0001.


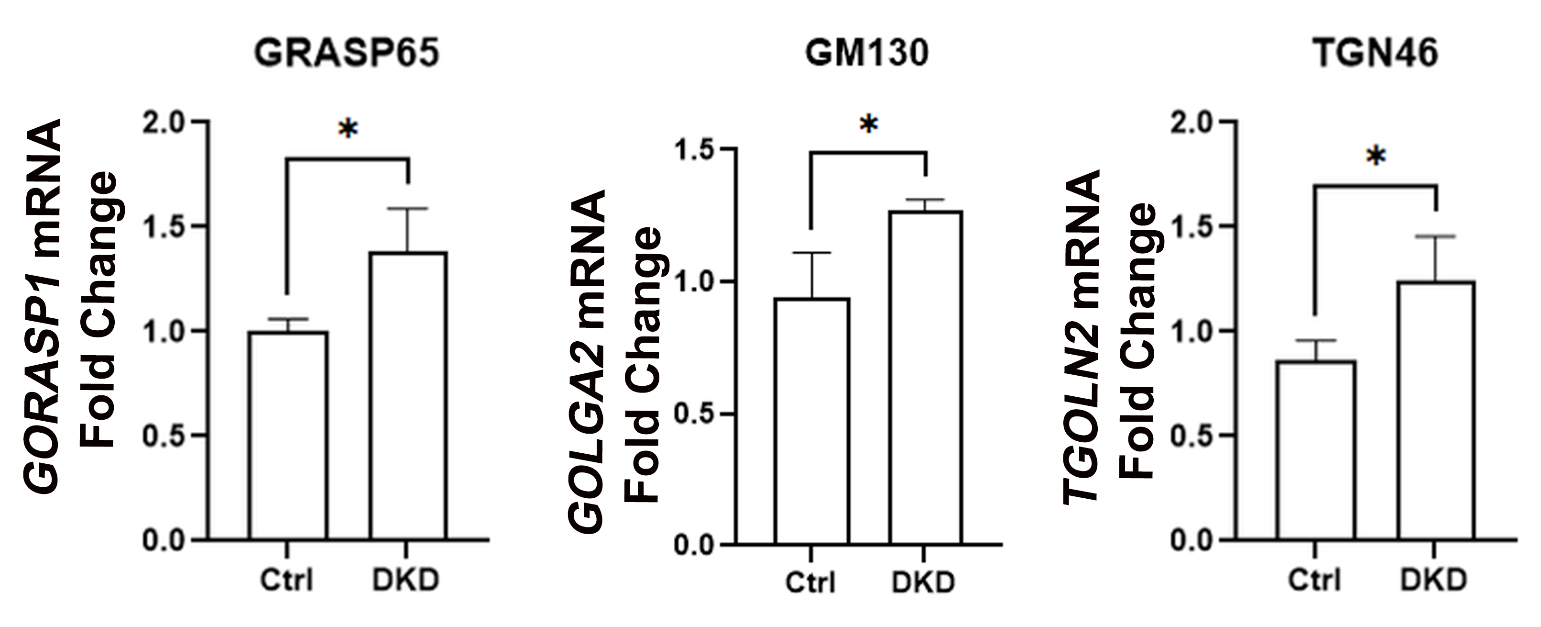


**Figure S3.** Quantification of fold change in mRNA expression of *GORASP1* (GRASP65), *GOLGA2* (GM130), and *TGOLN2* (TGN46) measured by qRT-PCR of RNA isolated from LNCaP cells treated with control or *STIM1* and *OSBPL5* siRNAs; Welch’s t test; * p≤0.05, mean ± SD.


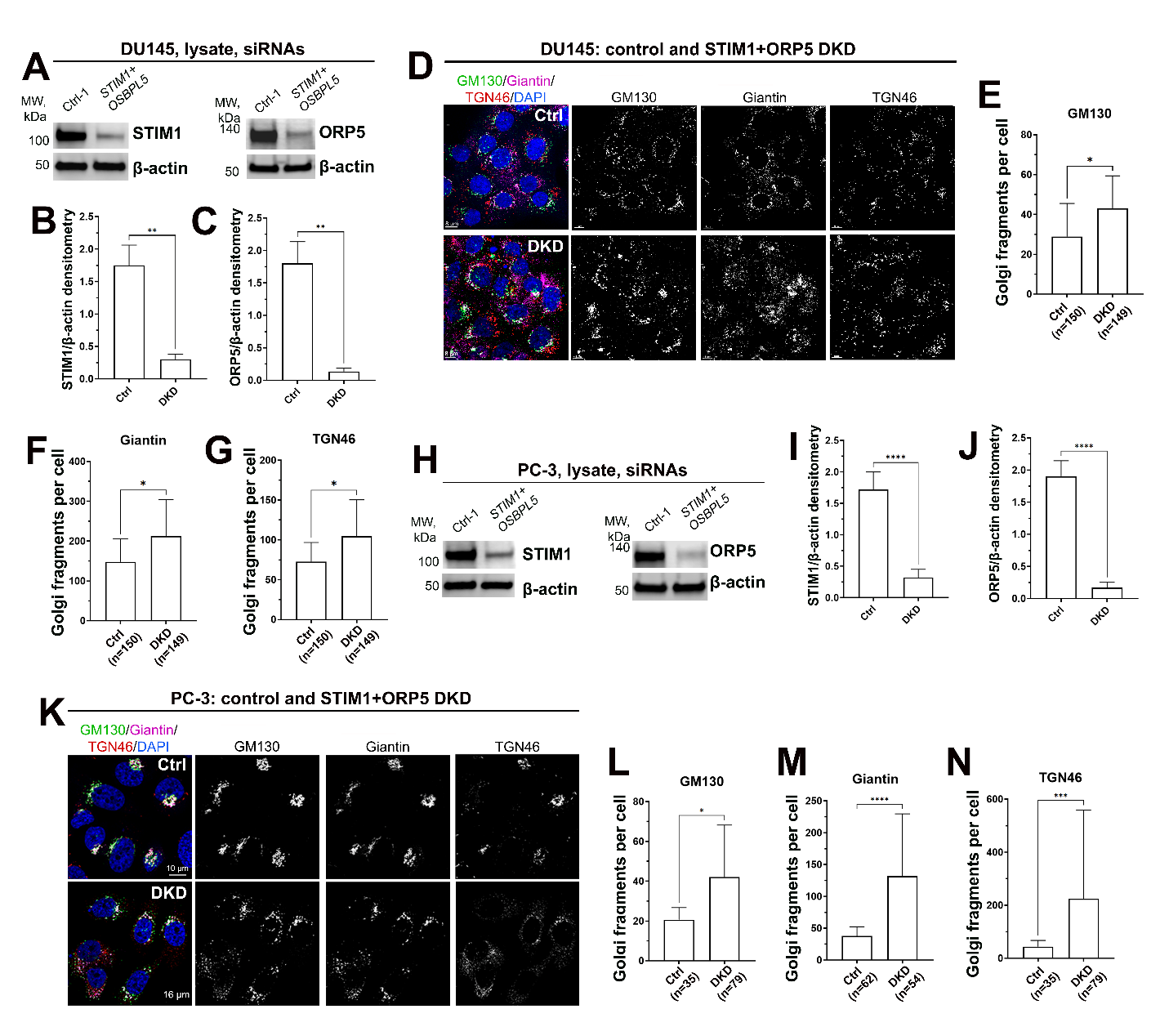


**Figure S4.** **(A)** STIM1 and ORP5 W-B of lysates from DU145 cells treated with control or *STIM1* and *OSBPL5* siRNAs. **(B-C)** Quantification of the densitometry for STIM1 (B) or ORP5 (C) in W-Bs represented by A; unpaired t test. **(D)** Representative Golgi IF images of DU145 cells treated with control or *STIM1* and *OSBPL5* siRNA stained for GM130 (green), Giantin (magenta), and TGN46 (red); bars, 8 μm. **(E-G)** Quantification of the number of Golgi fragments per cell for GM130 (E), Giantin (F), and TGN46 (G); Mann-Whitney test. **(H)** STIM1 and ORP5 W-B of lysates from PC-3 cells treated with control or *STIM1* and *OSBPL5* siRNAs. **(I-J)** Quantification of the densitometry for STIM1 (I) or ORP5 (J) relative to β-actin in W-Bs represented by H; unpaired t test. **(K)** Representative Golgi IF images of PC-3 cells treated with control or *STIM1* and *OSBPL5* siRNAs stained for GM130 (green), Giantin (magenta), and TGN46 (red); bars, 10 and 16 μm for Ctrl and DKD, respectively. **(L-N)** Quantification of the number of Golgi fragments per cell for GM130 (L), Giantin (M), and TGN46 (N); Mann-Whitney test. All graphs are representative of at least 3 independent experiments; n is the number of cells analyzed; * p≤0.05, ** p≤0.01, *** p≤0.001, **** p≤0.0001, mean ± SD.


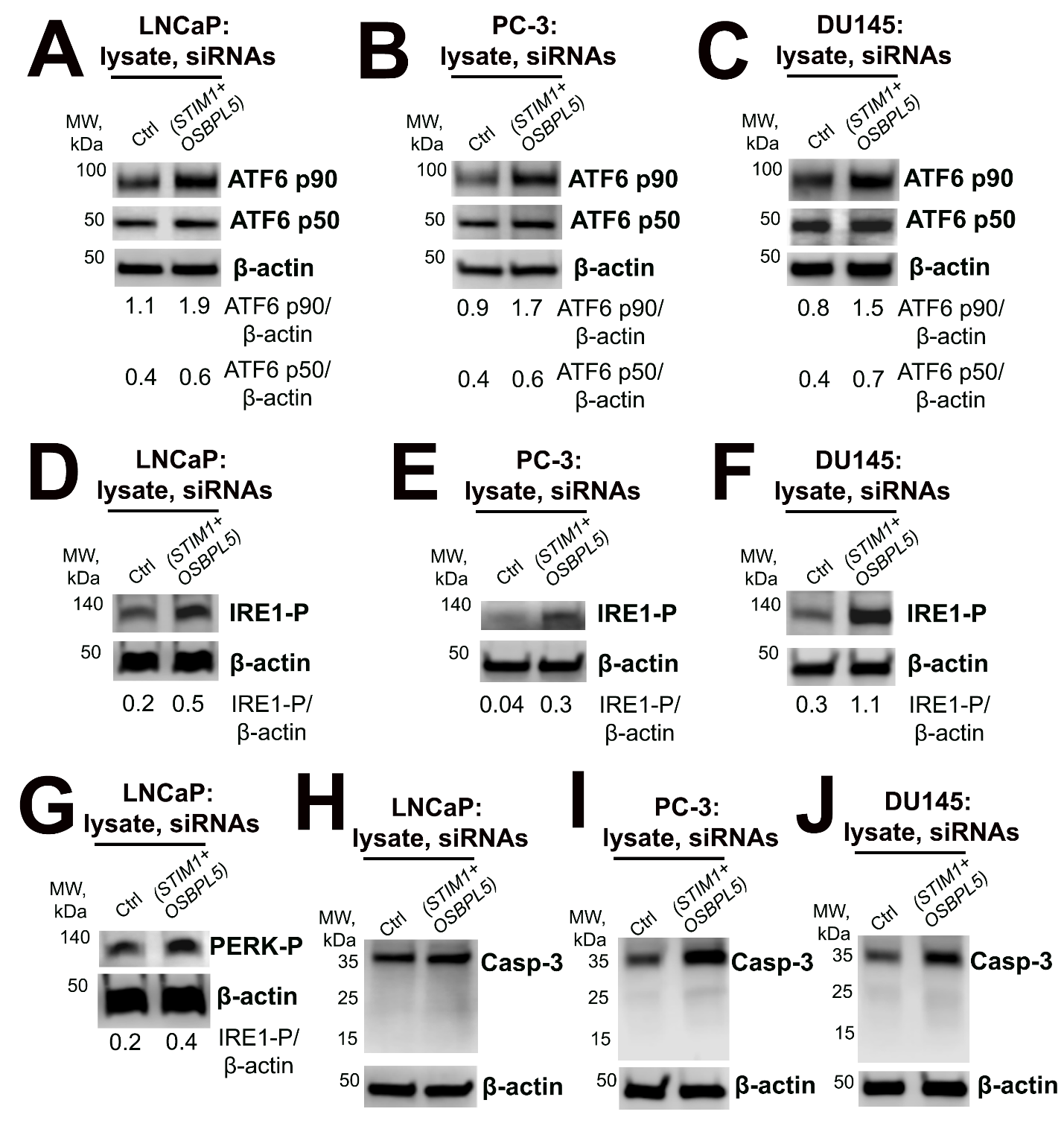


**Figure S5**. **(A-C)** ATF6α W-B of lysates from LNCaP (A), PC-3 (B), or DU145 (C) cells treated with Ctrl or *STIM1* and *OSBPL5* siRNAs. **(D-F)** IRE1-P W-B of lysates from LNCaP (D), PC-3 (E), or DU145 (F) cells treated with Ctrl or *STIM1* and *OSBPL5* siRNAs. **(G)** PERK-P W-B of lysates from LNCaP cells treated with Ctrl or *STIM1* and *OSBPL5* siRNAs. **(H-J)** Caspase-3 W-B of lysates from LNCaP (H), PC-3 (I), or DU145 (J) cells treated with Ctrl or *STIM1* and *OSBPL5* siRNAs. All results are representative of at least 3 independent experiments.


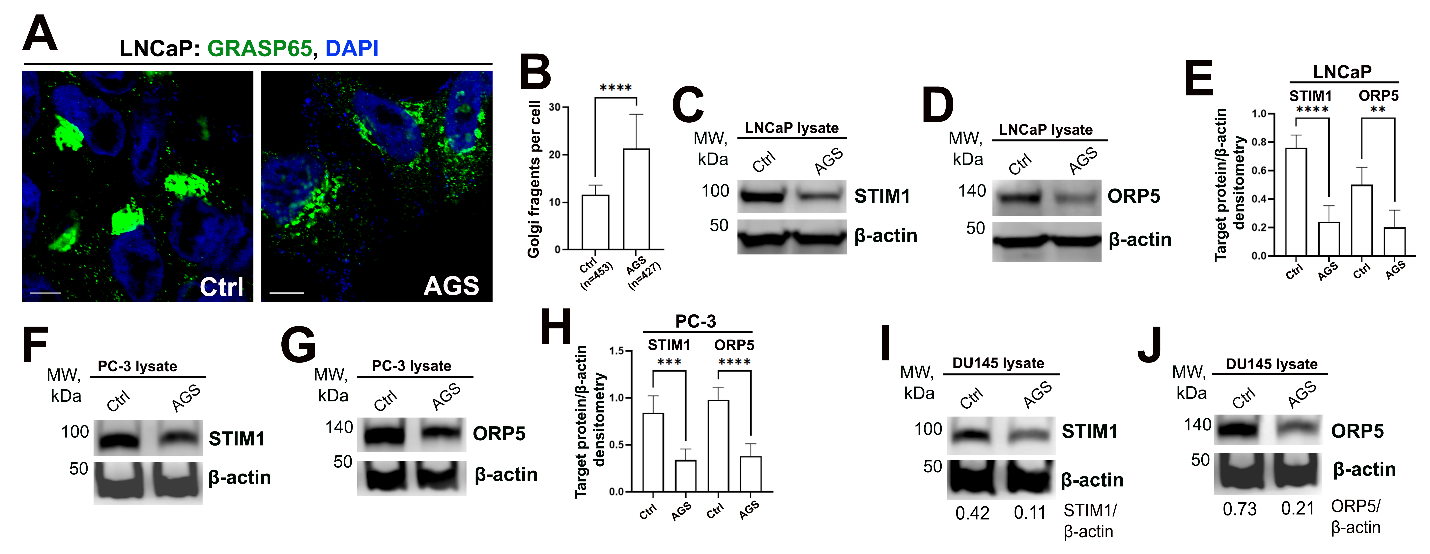


**Figure S6. (A)** Representative GRASP65 (green) IF images of LNCaP cells treated with AGS; bars, 10 μm. **(B)** Quantification of the number of Golgi fragments per cell from images in A; Welch’s t test, n is the number of cells counted. **(C and D)** STIM1 (C) and ORP5 (D) W-B of lysates from LNCaP cells treated with AGS. **(E)** Quantification of STIM1 and ORP5 densitometry for W-Bs represented by C and D; Welch’s t test. **(F and G)** STIM1 (F) and ORP5 (G) W-B of lysates from PC-3 cells treated with AGS. **(H)** Quantification of STIM1 and ORP5 densitometry for W-Bs represented by F and G; Welch’s t test. **(I and J)** STIM1 (I) and ORP5 (J) W-B of lysates from DU145 cells treated with AGS. For all graphs: ** p≤0.01, *** p≤0.001, **** p≤0.0001, mean ± SD. All results are representative of at least 3 independent experiments.


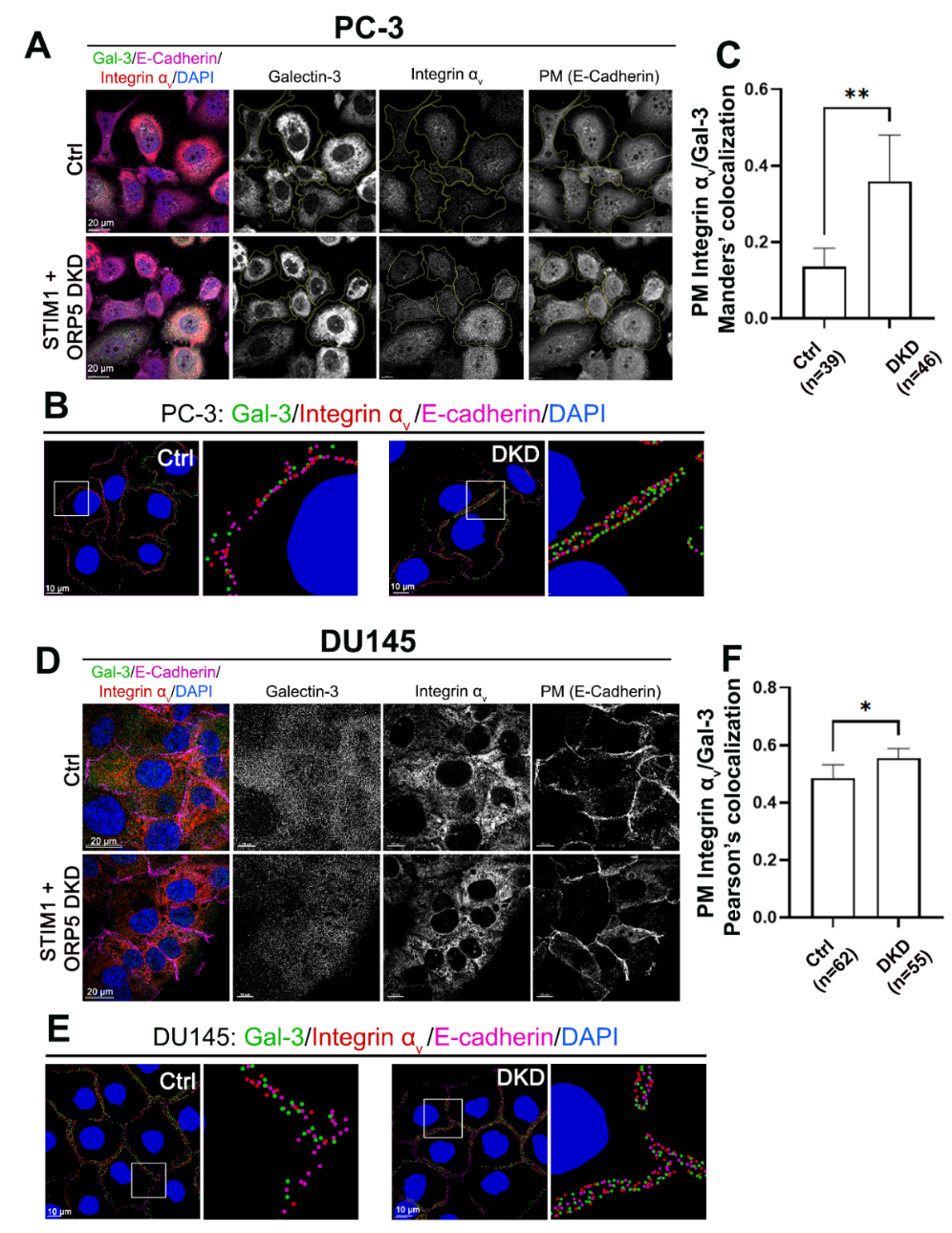


**Figure S7. (A)** Representative IF images of PC-3 cells treated with control or *STIM1* and *OSBPL5* siRNAs and stained for Gal-3 (green), Integrin α_v_ (red), and E-cadherin (magenta) as a membrane marker. The yellow outline demarks the PM as highlighted for analysis; bars, 20 μm. **(B)** Reconstructed images of Gal-3, Integrin α_v_, and E-Cadherin signal along the PM are represented by spots; bars, 10 μm. **(C)** Quantification of the Mander’s coefficient of colocalization for Integrin α_v_/Gal-3 in the highlighted PM region; Welch’s t test. Analysis was performed on the images shown in A, and represented by spots where red, green, and magenta overlap in B. **(D)** Representative IF images of DU145 cells treated with control or *STIM1* and *OSBPL5* siRNAs and stained for Gal-3 (green), Integrin α_v_ (red), and E-cadherin (magenta) as a membrane marker; bars, 20 μm. **(E)** Reconstructed images of Gal-3, Integrin α_v_, and E-Cadherin signal along the PM are represented by spots; bars, 10 μm. **(F)** Quantification of the Pearson’s *r* coefficient of colocalization for Integrin α_v_ and Gal-3 in the highlighted PM region. Analysis was performed on the images shown in D, and represented by spots where red, green, and magenta touch in E; Welch’s t test. In C and F, n is the number of cells analyzed. For all graphs: mean ± SD, * p≤0.05, ** p≤0.01.


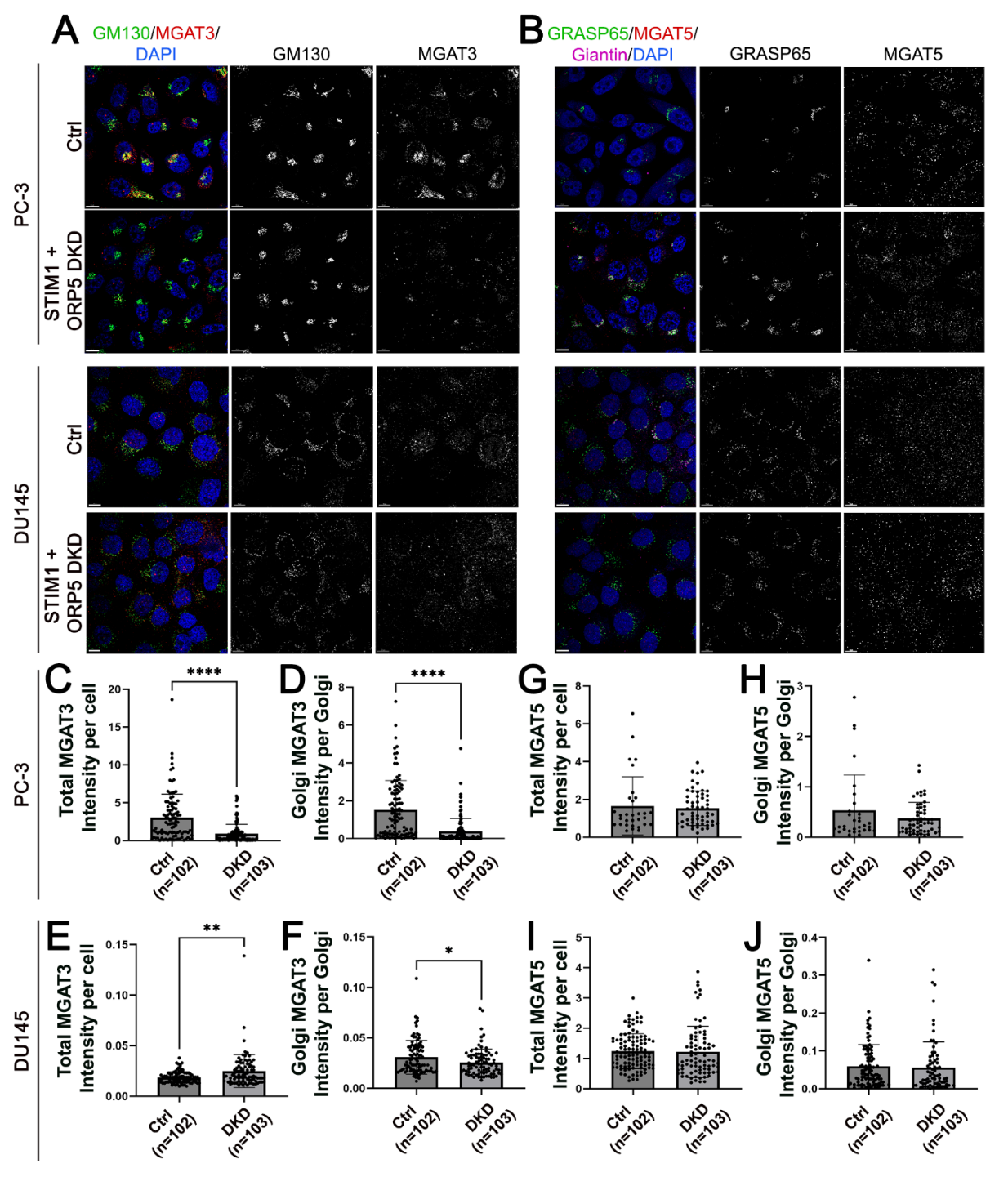


**Figure S8. (A-B)** Representative IF images of control or STIM1+ORP5 DKD PC-3 and DU145 cells stained for A: GM130 (green) and MGAT3 (red), or B: Giantin (magenta), GRASP65 (green) and MGAT5 (red). **(C and D)** Quantification of the MGAT3 total IF integrated density in the whole cell (C) or within the Golgi as defined by GM130 signal (D) in PC-3 images from A. **(E and F)** Quantification of the MGAT3 total IF integrated density in the whole cell (E) or within the Golgi as defined by GM130 signal (F) in DU145 images from A. **(G and H)** Quantification of the MGAT5 IF integrated density in the whole cell (G) or within the Golgi as defined by GRASP65 signal (H) in PC-3 images from B. **(I and J)** Quantification of the MGAT5 IF integrated density in the whole cell (I) or within the Golgi as defined by GRASP65 signal (J) in DU145 images from B; Mann-Whitney test, n is the number of cells counted. For all graphs: * p≤0.05, ** p≤0.01, **** p≤0.0001, mean ± SD.


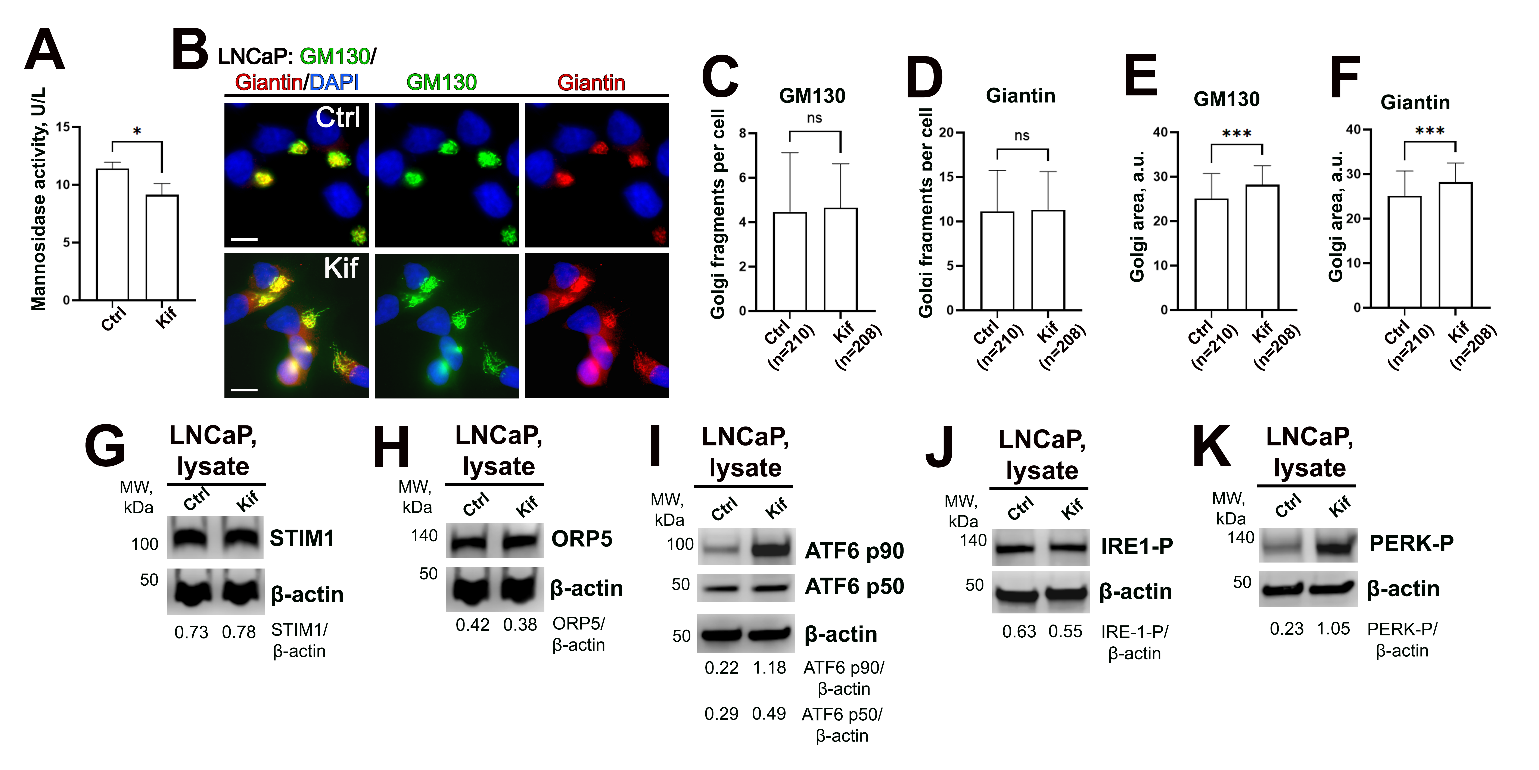


**Figure S9**. **(A)** Enzymatic activity of α-Mannosidase in LNCaP cells treated with 10 μg/ml Kifunensine (Kif) for 5 days compared to control cells; Welch’s t test. **(B)** Representative IF images of control or Kif-treated LNCaP cells, stained for GM130 (green) and Giantin (red); bars, 10 μm. **(C and D)** Quantification of Golgi fragments per cell from B for GM130 (C) and Giantin (D). **(E and F)** Quantification of Golgi area per cell from B based on GM130’s (E) or Giantin‘s IF signal (F). Mann-Whitney test for C-F. **(G-K)** STIM1 (G), ORP5 (H), ATF6 (I), IRE-1-P (J), and PERK-P (K) of the lysate samples from control or Kif-treated LNCaP cells. For all graphs: * p≤0.05, *** p≤0.001, n is the number of cells counted, mean ± SD.


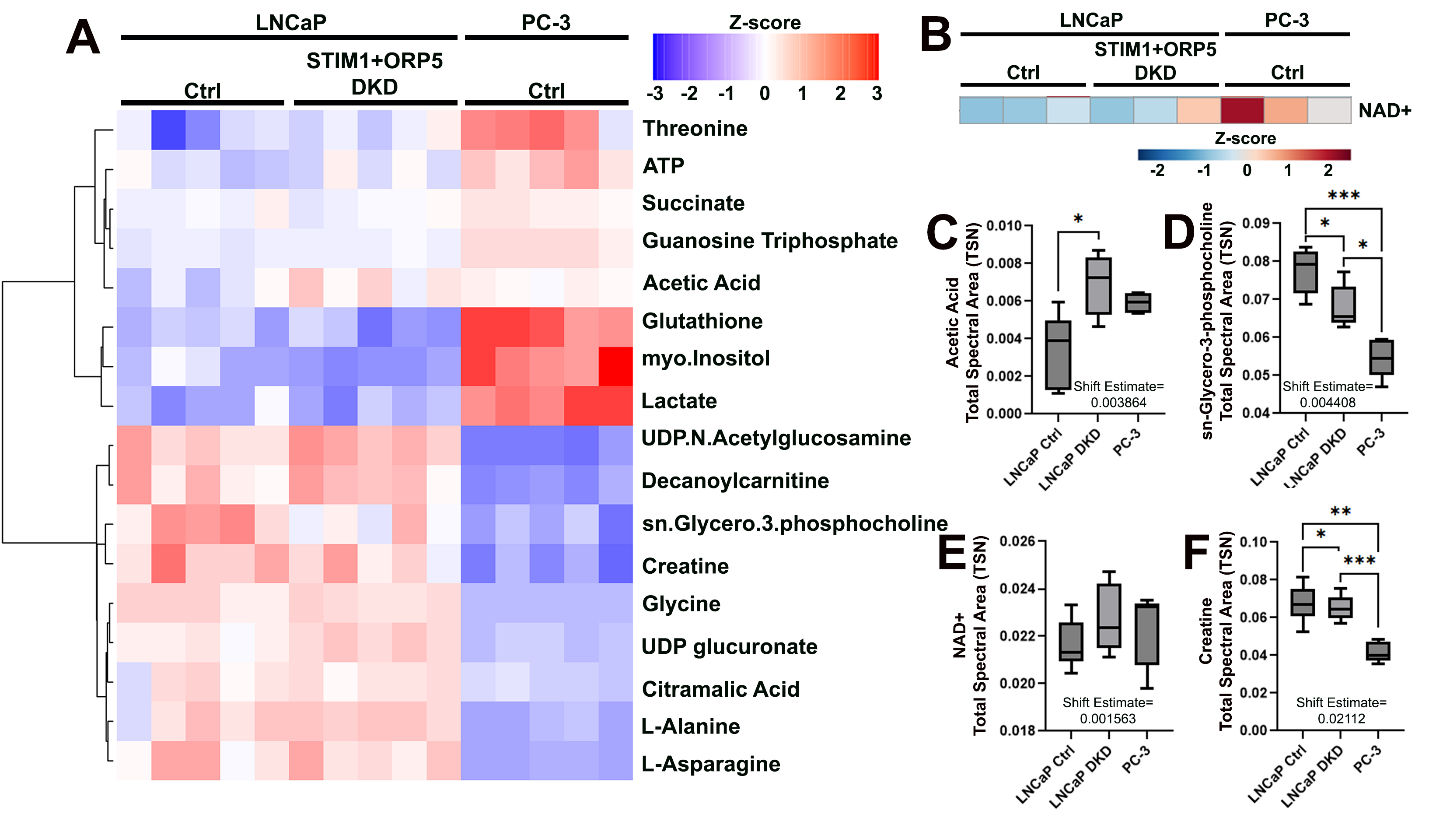


**Figure S10**. Metabolite analysis of Ctrl and STIM1+ORP5 DKD LNCaP cells compared to PC-3 cells. **(A-B)** Heatmaps and hierarchical clustering of metabolites. Each row displays the Z-score normalized abundance of the metabolite across the 3 groups. Red indicates metabolite accumulation, and blue represents depleted metabolite levels. Metabolites were analyzed from 5 samples (A) or 3 samples (B) per group. **(C-F)** Box plots of metabolites, demonstrating a positive shift in total spectral area for LNCaP DKD samples away from LNCaP Ctrl and toward PC-3: Acetic Acid (C), sn-Glycero-3-phosphocholine (D), NAD^+^ (E), and Creatine (F). Analyses were: Brown-Forsythe and Welch ANOVA and Welch’s t-test for C, D, E, and F. For all graphs: * p≤0.05, ** p≤0.01, *** p≤0.001; median ± SD.

**Table S1**

**
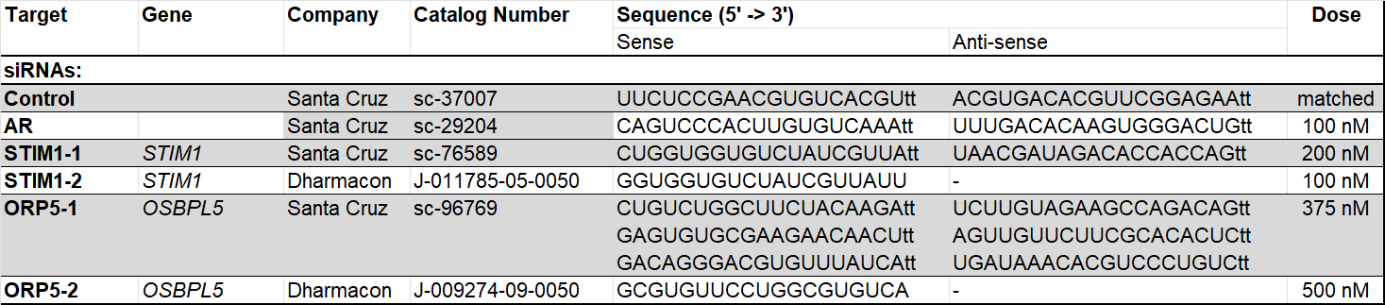
**

**Table S2**

| Target | Sequence 5'-3' | |
| --- | --- | --- |
|  | Forward Primer | Reverse Primer |
| *GAPDH* | ATGGGCAGCCGTTAGGAAAG | AGGAAAAGCATCACCCGGAG |
| *GM130* | TGGCGGCATCTCAGAATCAT | TGTTGGGAGAGTTGTCGCAG |
| *GRASP65* | CTCCACGGGGTGCAGGA | CTCGAGTGCCCAATGGTGAT |
| *KLK2* | GTCAGAGCCTGCCAAGATCA | GCGCAAGAACTCCTCTGGTT |
| *KLK3* | AGGTGACCAAGTTCATGCTGT | GGTGATCAGAATGACCCACGAG |
| *OSBPL5* | GGCACGGTAGGAGAAGCC | TTCTGAGGGGTGGAGGAAGG |
| *STIM1* | GCCAGAGCCTCAGCCATAG | AATTCGGCAAAACTCTGCTGC |
| *TGN46* | TCACAACAAGCGGAAGATCA | CGTTGGTAGTCACTGGCCTT |
| *TMPRSS2* | GGTGAAAGCGGGTGTGAGG | GGTCCAATAGCTGGTGGTGA |

**Supplemental Materials and Methods**

**Antibodies and Reagents**

Primary antibodies used were: **A)** rabbit polyclonal: OSBPL5 (NBP1-81403, Novus Biologicals); Androgen Receptor (ab74272, Abcam); Prostate Specific Antigen (ab53774); PERK (phospho T982) (ab192591); Caspase-3 (ab13847); TGN46 (ab16059); HDAC6-P (phospho S22) (ab61058); HSP90 alpha [ac Lys294] (NBP1-77944); MGAT3 (PA5-22212, ThermoFisher Scientific); MGAT5 (ab68595). **B)** rabbit monoclonal: Stromal interaction molecule 1 [EPR3414] (ab108994); Integrin alpha V [EPR16800] (ab179475); Galectin-3 (ab209344); ATF6 (ab227830); IRE1 (phospho S724) (ab288371); Sodium Potassium ATPase [EP1845Y] (ab76020); HDAC6 (D2E5) (7558S, Cell Signaling Technologies). **C)** mouse polyclonal: GM130 (ab169276). **D)** mouse monoclonal: β-actin (A2228, Sigma-Aldrich); E-Cadherin (ab1416); GRASP65 (sc-365434, Santa Cruz Biotechnology); E-Syt1 (sc-514488, Santa Cruz Biotechnology). **E)** goat polyclonal: Calreticulin (ab4109); E-Cadherin (AF748, Bio-Techne Corporation). **F)** sheep polyclonal: GOLGB1/Giantin (AF8159, R&D Systems). Secondary antibodies were: a) Jackson ImmunoResearch: HRP-conjugated donkey anti-rabbit (711-035-152) and donkey anti-mouse (715-035-151) for W-B; b) Invitrogen: Alexa Fluor 488-conjugated donkey anti-mouse (A-21202), Alexa Fluor 594-conjugated donkey anti-rabbit (A-21207), Alexa Fluor 647-conjugated donkey anti-goat (A-21447), Jackson ImmunoResearch: Alexa Fluor 647 AffiniPure donkey anti-sheep (713-605-147) for IF. N-ethylmaleimide (NEM, Thermo Fisher Scientific) was applied to lysates at a final concentration of 2 mM. BioWorld Allium sativum Lectin (ASA) (21510973-1) conjugated to 1-5nm gold beads was used for EM detection of high-mannose glycans.

**siRNA transfection**

Knockdown of proteins by siRNA was carried out using Lipofectamine RNAi MAX Transfection Reagent (Thermo Fisher Scientific, 13778150) following the manufacturer‘s recommendations. See **Table S1** for sequences, concentrations, and catalog numbers. Cells were incubated with antibiotic-free media for 24 hours before a 10-24 hour incubation with siRNA media. Most siRNAs were applied for 24 hours, then recollected and replaced with antibiotic-free media for an additional 48 hours before collecting cells for sample preparation. For siRNA targets with high expression levels, the siRNA treatment is repeated for another 24 hours on the third day (after 24 hours of incubation in antibiotic-free media).

**Fluo-4,AM Live Calcium Staining**

Calcium influx was measured with the cell-permeant dye Fluo-4, AM (F14201; Thermo Fisher Scientific), following the manufacturer’s instructions. Cells were incubated with 5 µM Fluo-4, AM, and Hoechst (1:3000 dilution) in 1 mL of complete RPMI medium for 30 minutes at 37 °C. After three washes with pre-warmed (37 °C) 1× PBS, live cells were imaged using a 20× objective on an EVOS AMF-4300 fluorescence microscope. To directly evaluate Ca²⁺ influx rather than static fluorescence differences, we monitored time-resolved Fluo-4 signals over 30 minutes. For both control and STIM1/ORP5-DKD LNCaP cells, the lowest fluorescence observed at 0 minutes was used as the baseline (F₀). Each later time point was normalized by subtracting this baseline (ΔF = F(t) − F₀). This method accounts for differences in dye loading and resting Ca²⁺ levels, ensuring the measurement reflects influx. Afterwards, the cells were fixed with 4% formaldehyde for 2 minutes at room temperature, rinsed with PBS, and mounted with an aqueous mounting medium. Fixed samples were imaged the following day using a 60× objective. These fixed samples were solely used to enhance visualization of the GFP signal in separate imaging experiments, providing representative images for the manuscript.

**RNA Isolation**

RNA was isolated according to the manufacturer's instructions using the RNeasy Mini Kit (Qiagen, 74004). Briefly, cells were collected, lysed, and homogenized by passing through a 22-G needle. The lysate was mixed with 70% ethanol and loaded onto the RNeasy spin column. After washing, the optional on-column DNase I digestion was performed with the RNase-Free DNase Set (Qiagen, 79254). The digested DNA was then washed away, and the RNA was eluted in water and quantified using a NanoDrop Lite Spectrophotometer (Thermo Fisher Scientific).

**One-Step Quantitative Real-Time Polymerase Chain Reaction (qRT-PCR)**

The relative amount of mRNA was assessed with the Luna Universal One-Step RT-qPCR Kit (New England BioLabs, Inc., E3005L) and a one-step PCR on either a StepOnePlus Real-Time PCR System (Thermo Fisher Scientific) or the BioRad C1000 Touch Thermalcycler with the CFX96 Real-Time System (BioRad, 1841100 and 1845097, respectively). Each reaction contained the recommended ratios of 500 ng RNA, 10 μL 2x Reaction Mix, 1 μL 20x Enzyme Mix, 0.8 μL 10 μM forward primer, 0.8 μL 10 μM reverse primer, and water to a final volume of 20 μL; primers are listed in **Table S2**. The thermocycler settings were 55°C for 10 minutes, 95°C for 1 minute, [95°C for 10 seconds, 60°C for 30 seconds, plate read] x40 cycles, melt curve (60-90°C), as recommended for the Luna kit. The relative gene expression was quantified using the 2^-ΔΔC^_T_ method^1^ with the reference gene being GAPDH.

**Determination of α-Mannosidase Activity**

To inhibit α-Mannosidase activity, cells were treated with the α-Mannosidase inhibitor Kifunensine (Cayman Chemical, 109944-15-2) at 10 μg/ml for 5 days. Control cells receive an appropriate amount of molecular-grade water. The α-Mannosidase activity assay kit (Sigma-Aldrich, MAK318) was used according to the manufacturer's recommendations. In short, an equal number of cells were homogenized for each sample with a cold 50 mM potassium phosphate buffer, pH 7.5.. After centrifugation at 10,000 *xg* for 15 minutes, the supernatant was collected as the sample. In each well, 10 μL of sample and 90 μL of substrate buffer were incubated for 10 minutes at 25˚C. Then 100 μL of stop reagent was added to each well. Finally, the absorbance at 405 nm was measured. The activity was calculated by the following formula:

$$Activity (U/L)= \frac{{OD}_{SAMPLE}-{OD}_{BLANK}}{10min\times slope of standard curve}\times\frac{200\mu L}{10\mu L}$$

**Plasma Membrane Protein Isolation**

The PM-localized proteins were labelled while the membrane remained intact with 0.25 mg/mL EZ-Link™ Sulfo-NHS-Biotin (Thermo Fisher Scientific, 21217) and isolated using the Pierce™ Cell Surface Protein Isolation kit (Thermo Fisher Scientific, 89881). After incubation with biotin, cells were washed, pelleted, and lysed. The lysates were incubated with streptavidin beads to capture biotinylated PM proteins, while internal proteins flowed through into the recovered lysate. The streptavidin-biotin-bound proteins were then eluted from the beads using 1M dithiothreitol (DTT; Acros Organics, 16568-0050). Samples in DTT were used directly in W-B assays or reconstituted in 1X running buffer using Amicon Ultracel 30K centrifugal filters (Millipore, UFC503024) for use in enzymatic digestion and glycoproteomic analysis.

**Lectin-Affinity Isolation**

Lysates or PM samples were collected as usual; PM samples were reconstituted in 1X running buffer prior to use. Protein concentrations were normalized with 1X running buffer to 2-20mg/mL and then diluted 1:1 in the appropriate binding buffer [PHA-L (21511265-3, GlycoMatrix), ConA (21510892-3, GlycoMatrix), or ASA (21511254-3, GlycoMatrix)]. An aliquot of diluted lysate was kept back as “Input” for the W-B. Columns were prepared with 1mL of bead slurry for the appropriate lectin-bound beads [Phaseolus vulgaris Lectin (PHA-L) - Separopore 4B (30330038-3), ConA (20120057-3, GlycoMatrix), or ASA (21510977-2, GlycoMatrix)] in each Pierce Spin Column (69725, Thermo Scientific) and washed 5 times with binding buffer. Diluted lysates were applied to washed columns and incubated overnight at 4°C with rotation. Columns were centrifuged at 1000 × g to recover any unbound proteins from the diluted lysate, and the resulting fraction was stored as the recovery lysate. Then, the columns were washed with binding buffer 5-10 times until the flow-through contained undetectable levels of protein, as determined by NanoDrop A280 analysis. The beads were then incubated overnight at 4°C with the appropriate elution buffer [PHA-L (21511303-3, GlycoMatrix), ConA (21511337-3, GlycoMatrix), or ASA (21511280-3, GlycoMatrix)]. The final flow-through after centrifugation at 1000 × g was retained as the eluate and contained any protein that bound the lectin’s target glycan. In cases of poor elution, the beads were also boiled and incubated with 10% β-mercaptoethanol in 2x loading dye at 95°C for 10 minutes, and the flow-through was then loaded onto a W-B gel.

**Endoglycosidase H Digestion**

Lysate and PM samples were incubated with Endoglycosidase H (P0702L, New England BioLabs, Inc.) according to the manufacturer‘s recommendations to cleave the chitobiose core of high-mannose glycans. In short, samples were normalized to equal protein concentrations, yielding a total sample amount of up to 20 μg of protein per reaction. Each sample was combined with 1 μL of Glycoprotein Denaturing Buffer (10X) and nuclease-free water to a final volume of 10 μL per reaction. After heating this mixture for 10 minutes at 100°C, 2 μL of GlycoBuffer 3 (10X), 1-5 μL of EndoH, and enough water to bring the total volume to 20 μL were added to the reaction. The reaction was then incubated at 37°C for 1 hour. Samples were then prepared for W-B.

**Neuraminidase Digestion**

Lysates were incubated with α2-3,6,8,9 Neuraminidase A (P0722L, New England BioLabs, Inc.) according to the manufacturer's recommendations to cleave linear and branched, non-reducing terminal sialic acid residues. Samples were normalized to 1 μg of sample protein and diluted to a final volume of 9 μL with water. Then, 1 μL of GlycoBuffer 1 (10X) and 1 μL of α2-3,6,8,9 Neuraminidase A were added. The reaction mixture was then incubated for 1 hour at 37°C, and samples were prepared for W-B.

**Metabolomic Analysis**

Metabolites were isolated and analyzed as described previously^2^. Specifically, after treatment, cells were rinsed with PBS (pre-warmed to 37 °C) followed by one quick rinse with sterile water. Then, cells were incubated at -80°C for 15 minutes with 80% methanol in sterile water pre-chilled to -80°C. Cells were then collected by scraping and centrifuged at full speed for 5 minutes at 4°C. The supernatant was then collected into a fresh tube, and the pellet was washed with sterile water. After centrifuging at full speed for 5 minutes at 4°C, the supernatant was added to the previous supernatant and submitted for analysis by the Systems Biology Core at the University of Nebraska at Lincoln. There, supernatants were dried by vacuum evaporation (SpeedVac Plus, Savant, Thermo Scientific, Waltham, MA) followed by freeze-drying (Labconco, Kansas City, MO).

Cell lysate extracts were reconstituted in 200 μL of 50 mM phosphate buffer at pH 7.2, containing 50 μM 3-(tetramethylsilane) propionic acid-2,2,3,3-D4 (TMSP-D4) added as a chemical shift reference made in 99.8% D_2_O. Lysate and medium samples were resuspended by vortexing and centrifuged at 14000 rpm for 30 min to pellet any insoluble particulates and tracer impurities. Lysate and media samples were transferred into 3 mm tubes to obtain higher concentrations of metabolite extracts and improve the sensitivity of low-concentration metabolites during NMR experiments.

All data were collected on a Bruker Avance III-HD 700 MHz spectrometer equipped with a quadruple resonance QCI-P cryoprobe (1H, 13C, 15N, and 31P with 2H lock and decoupling) and an automated sample changer. For media samples, the 1D 1H NMR spectra were collected at 298 K with 32K data points, 128 scans, 4 dummy scans, and a spectral width of 9090 Hz using an excitation sculpting pulse sequence. Fifty percent nonuniformly sampled (NUS) deterministic burst augmented gap schedule. TopSpin 3.6.2 was an instrument monitoring software.

Data preprocessing was performed using the Chenomx NMR Suite 8.0 software. The preprocessing steps include Fourier transformation, phase correction, baseline correction, shim correction, line broadening, and calibration with the TMSP. For metabolite identification, the chemical shifts were compared with the HMBD and Chenomx metabolite libraries. The dataset of concentrations of identified metabolites was used for further statistical analysis. Before analysis, the data were normalized by sum and Pareto-scaled. The PCA, OPLS-DA, and the heatmap diagrams were generated using MetaboAnalyst 6.0. Box plots were developed using R. The differences between groups were analyzed using the Brown-Forsythe and Welch ANOVA tests or ANOVA with Kruskal-Wallis. The shift of LNCaP DKD towards PC-3 was then tested using a directional shift analysis, which yields an estimated value greater than 0 if the DKD group is significantly closer to PC-3 than to Ctrl with a p < 0.05, an estimated value of 0 when DKD is equidistant between PC-3 and LNCaP Ctrl, and an estimated value less than 0 when the DKD group is closer to LNCaP Ctrl than to PC-3.

**Immunofluorescent Staining**

For immunofluorescent staining of target proteins, cells were fixed with 4% formaldehyde in PBS and permeabilized with 0.2% Triton X-100 in PBS. The cells were then blocked with 1% donkey serum to prevent non-specific antibody binding. Primary antibodies were applied for 2 hours at room temperature at a 1:50 dilution of 1 mg/mL antibody in 1% donkey serum in PBS-T. After washing, secondary antibodies were applied at a 1:200 dilution in 1% donkey serum in PBS-T for 1 hour at room temperature. Following final PBS-T washes, the cover slips were mounted on slides using ProLong Gold antifade reagent with DAPI (P36935, Invitrogen).

Slides were imaged using the EVOS M3000 with a Plan-Apochromat 63x/1.4 Oil DIC M27 objective on the Zeiss LSM800 Microscope with Airyscan at the University of Nebraska Medical Center Advanced Microscopy Core Facility, or the ELYRA. All slides in an experiment were imaged with consistent microscope settings and analyzed with the same analysis parameters. Detection wavelengths were 410-470 nm for blue, 410-546 nm for green, 572-617 nm for red, and 656-700 nm for far red.

Protein immunofluorescent intensity was analyzed as integrated density in Fiji using the measure tool. All CZI images were converted to TIF files using Imaris 9.1.2 (Bitplane Scientific) or Zen Blue (Zeiss) software. Images were opened in Fiji, converted to 8-bit, and thresholded to a single threshold for the entire experiment. The integrated density was then recorded for the image or selected area. For plasma membrane protein intensity, the PM marker was used to outline the PM's outer edge with a freeform ROI. The area within this ROI was recorded as the outer area. Then the ROI was reduced by 10 pixels using the enlarge tool. The ROI area was recorded as the inner area. The outer area minus the inner area determined the area of the PM. All signals outside the outer ROI and inside the inner ROI were removed. The remaining signal was then measured as the PM signal. The PM intensity was then normalized to the PM area (PM intensity/PM Area).

Colocalization was quantified using the JACoP plugin in Fiji^3^, keeping the input channel the same for images A and B throughout the experiment. Images were prepared by removing non-specific signal, incomplete cells, or the PM, as described above. The prepared images were input to image A and B, and the outputs were recorded: Pearson’s *r* coefficient, M1 (Mander’s coefficient A), and M2 (Mander’s coefficient B).

**Immunohistochemistry (IHC)**

IHC was performed using the Rabbit-specific HRP/DAB (ABC) Detection IHC Kit (Abcam, ab64261) or the TripleStain IHC Kit (M&R&G on human tissue; DAB, AP/Red & HRP/Green) (Abcam, ab183290) according to the manufacturer's recommendations. In brief, tissues were deparaffinized and rehydrated using the following regimen: 2 x 10-minute incubations in xylene, followed by 5 minutes each in 100%, 95%, 70%, and 50% ethanol. Heat-induced epitope retrieval was performed by boiling the slides in sodium citrate buffer at pH 6.0 for 20 minutes. Slides were then cooled under running tap water and incubated for 20 minutes with 0.2% Triton X-100 in TBS. Then, the hydrogen peroxide block buffer was applied for 10 minutes, followed by the protein block buffer for 10 minutes. The slides were then incubated with primary antibodies diluted 1:50 in protein block buffer for 2 hours at 37°C. Slides were then washed and incubated with Goat-HRP and Rabbit-AP polymers for 30 minutes at 37°C. After washing, slides were incubated with DAB for 2 minutes at room temperature, then washed immediately. Subsequent incubation with permanent red solution is performed until the stain develops. Then, the slides are washed with water and incubated for 30 minutes at room temperature with a triple stain blocker. Finally, slides are counterstained with hematoxylin, bluing reagent, and emerald chromogen in succession. The slides are then washed with tap water and air-dried. The rehydration and deparaffinization regime is then performed in reverse: 1 minute each for 70%, 95%, and 100% ethanol, followed by 2 more rounds of 100% ethanol, and a 10-second dip in xylene. Slides are then air-dried, and cover slips are mounted with ProLong Gold antifade reagent (P36930, Invitrogen). All IHC slides were digitized using a Leica Aperio CS2 whole-slide scanner equipped with a 20× Plan Apo objective (numerical aperture 0.75). Images were acquired at a native spatial sampling of 0.50 μm/pixel, corresponding to an effective lateral optical resolution of approximately 0.7-0.8 μm, consistent with the diffraction limit for a 0.75 NA objective under brightfield illumination. This resolution is standard and sufficient for discriminating plasma membrane vs. cytoplasmic signals in chromogenic IHC. Specifically, the plasma membrane H-score was assessed using the widely validated Multiplex IHC v3.1.4 Cell Surface algorithm in HALO v3.4 (Indica Labs, Inc.) for quantitative analysis of membrane biomarkers.

**Immunogold Electron Microscopy**

Cells were plated in 35-mm glass-bottom MatTek dishes (P35-1.5-14-C-GRID, MatTek) to achieve approximately 40% confluence, ensuring minimal cell clustering. After the specified treatment, cells were washed and fixed with a solution of 0.1% glutaraldehyde and 4% paraformaldehyde in 0.1M phosphate buffer at pH 7.4. Next, aldehydes were blocked with a 50 mM glycine solution in 0.1 M phosphate buffer. Permeabilization was performed with 0.25% saponin (Millipore, 558255) in 0.1 M phosphate buffer. Cells were then blocked with 0.1M phosphate buffer containing 0.02% saponin, 1% BSA (Tocris Bioscience, 5217), 0.2% fish gelatin (Bioworld, 21761058), and 5% goat serum (Jackson ImmunoResearch, 005-000-121). Primary antibodies were applied at a 1:50 dilution in blocking buffer overnight at 4°C, followed by secondary antibodies diluted 1:20 and applied at room temperature for 1 hour. Washing steps included: 0.02% saponin/1% BSA/0.1M phosphate buffer; 0.02% saponin/0.1M phosphate buffer; 0.1M phosphate buffer; 1% glutaraldehyde/0.1M phosphate buffer; 50 mM glycine/0.1M phosphate buffer; and 0.1M phosphate buffer. Fluorescent signals were verified, and cells were then stored in 0.1M phosphate buffer for shipping to the University of Texas Southwestern Medical Center, where collaborators rinsed the cells with water and 0.1M sodium cacodylate buffer. Post-fixation involved treatment with 1% osmium tetroxide and 0.8% K3[Fe(CN)6] in 0.1M cacodylate buffer. Cells were then stained en bloc with 2% aqueous uranyl acetate and dehydrated through a series of increasing ethanol concentrations. Subsequently, cells were infiltrated with Embed-812 resin (Electron Microscopy Sciences, 14120) and polymerized. The blocks were sectioned with a Leica Ultracut UCT ultramicrotome (Leica Microsystems) and collected on copper grids. Sections were imaged using a JEOL 1400 Plus (JEOL) with a LaB6 source at 120 kV.

**Mass Spectrometry N-glycan analysis**

Briefly, N-glycans were prepared from samples using the glyPAQ kit (beta version; ProtiFi, Fairport, NY) per the manufacturer’s instructions. Reduced N-glycans were injected onto an Ultimate 3000 UHPLC system capillary pump coupled to an Orbitrap Eclipse mass spectrometer (ThermoFisher Scientific, Waltham, MA) as described in Wojtkiewicz et al^4^. The self-made microcolumn was packed with Hypercarb PGC (180 μm × 100 mm, 3 μm Hypercarb, Thermo Fisher Scientific). Mobile phase A was comprised of 10 mM ammonium bicarbonate, and mobile phase B was 10 mM ammonium bicarbonate in 60% (v/v) acetonitrile. Samples were injected directly onto the column with 2 μL/min flow in 2% buffer B for 3 minutes with a post-column makeup flow of acetonitrile at 3 μL/min. Ionization was achieved using an MnESI source (Newomics, Berkeley, CA) and a Microfabricated Monolithic Multi-nozzle (M3) 5-nozzle emitter. MS spectra were acquired with a m/z range of 500–2000; AGC target set to 8 × 10^5^ and maximum injection time to 200 ms. Fragments of the most abundant precursors were generated using collision-induced dissociation at 33%, isolated within 2 ppm, for a maximum scan time of 4 seconds. MS/MS AGC target was set to 2 × 10^4^ and maximum injection time was set to 200 ms.

For glycan composition analysis, raw files were processed using GlycReSoft version 0.4.25. Briefly, a reduced glycan search space containing feasible human N-glycans, limited to 26 monosaccharides, was used as provided in the software. Mass matching error tolerance was set to 10 ppm, and peak grouping error tolerance was set to 15 ppm. No additional model features were chosen. These processed results are exported as *_glycan-chromatograms_found.csv files, where each row represents a unique glycan composition (e.g., Fuc:1; Hex:8; HexNAc:3; Neu5Ac:1) along with its quantitative and spectral parameters.

**Plasmid DNA extraction and transfection**

NeonGreen-C1-ORP5 (Addgene plasmid #220071)^5^ and STIM1-EGFP (Addgene plasmid #210370)^6^ were used for bacterial amplification. Bacterial glycerol stabs were streaked onto LB agar plates containing kanamycin (50 µg/mL) and incubated overnight at 37 °C. A single isolated colony was inoculated in 5mL of LB broth (containing kanamycin at a concentration of 50 µg/mL)and incubated for 8 hrs with shaking at 200rpm. Subsequently, the primary culture was inoculated into 300 mL of LB broth supplemented with kanamycin (final volume containing 150 µL of a 100 mg/mL stock) and cultured overnight at 37 °C with shaking at 200 rpm. Plasmid DNA was purified using the QIAGEN Plasmid Maxi Kit (Cat. No. 12162) according to the manufacturer’s protocol. The bacterial culture was centrifuged at 6,000 rpm for 15 min at 4 °C, and the supernatant was discarded. The pellet was resuspended in 10 mL of Buffer P1 (resuspension buffer). Cell lysis was achieved by adding 10 mL of Buffer P2 and gently inverting the tube 5times. Neutralization was performed by adding 10 mL of Buffer P3 and gently inverting to mix. The lysate was centrifuged at >20,000 × g for 30 min to remove debris, and the clear supernatant was transferred to a new tube and centrifuged again for 15 min at the same speed. A QIAGEN-tip 500 column was equilibrated with 10 mL of Buffer QBT and allowed to drain by gravity flow. The clarified lysate was loaded onto the column and permitted to enter the resin by gravity. The column was then washed twice with Buffer QC, and plasmid DNA was eluted using Buffer QF. DNA was precipitated by adding 10.5 mL of room-temperature isopropanol and centrifuging at 15,000 × *g* for 10 min. The resulting pellet was washed with 5 mL of 70% ethanol, centrifuged again, air-dried briefly, and dissolved in 100 µL of molecular-biology–grade water. Plasmid concentration and purity were assessed using a Nanodrop spectrophotometer (ThermoFisher Scientific).

One day before transfection, PC-3, LNCaP, and DU145 cells were seeded at a density of 0.5 million cells per well in a 6-well plate. After 24 hrs., cells, which had reached approximately 70-80% confluency, were transduced. The ensuing transfection protocol is for 1 transfection reaction, i.e., 1 well. Two sterile tubes were labelled as A and B. In both tubes, 125µL of Gibco™ Opti-MEM™ I Reduced Serum Medium (Catalog number: 31985070) was added. Next, 5 µL of Lipofectamine 3000 was added to tube A and mixed gently by pipetting up and down. In tube B, 5 µg of DNA (2.5 µg of each plasmid, i.e., NeonGreen-C1-ORP5 and STIM1-EGFP) and 10 µL of P3000 reagent were added and mixed well by pipetting up and down. The contents of tube B were added to tube A and mixed gently by pipetting up and down. It was then incubated at room temperature for 30 minutes. Meanwhile, spent media was removed from cells and replaced with 1mL of Opti-MEM. After incubation, 250 µL of complexes was added to the well dropwise and mixed by gently rocking the plate back, forth and sideways once. The transfection medium was replaced with complete growth medium after 12 hours, and the cells were analyzed by fluorescence microscopy after 48 hours for GFP expression as a measure of transfection efficiency.

**Adhesion assay**

Cell adhesion was evaluated using a CytoSelect™ 48-Well Cell Adhesion Assay ECM Plate (Catalogue # CBA-070). Briefly, the ECM-coated plate was brought to room temperature for 10 minutes under sterile conditions. Cells were harvested, resuspended in serum-free medium at a concentration of 0.1×10⁶ cells/mL, and gently mixed. A total of 150 µL of the cell suspension was added to each well, with bovine serum albumin (BSA)-coated wells serving as negative controls. The plates were incubated for 90 minutes at 37 °C in a humidified CO₂ incubator to allow cell attachment. After incubation, non-adherent cells were removed by aspiration, and each well was washed 5 times with 250 µL of phosphate-buffered saline (PBS), taking care not to let the wells dry out. Adherent cells were fixed and stained by adding 200 µL of Cell Stain Solution to each well and incubating for 10 minutes at room temperature. The stain was then removed, and the wells were gently washed five times with 500 µL of deionized water. After the final wash, the wells were air-dried completely. For quantification, 200 µL of Extraction Solution was added to each well and incubated for 10 minutes on an orbital shaker. Next, 150 µL of the extracted solution was transferred to a 96-well microplate, and absorbance was measured at 560 nm using a microplate reader. Relative adhesion was expressed as optical density (OD₅₆₀) values normalized to control wells.

**Supplemental References**

1 Livak, K. J. & Schmittgen, T. D. Analysis of relative gene expression data using real-time quantitative PCR and the 2(-Delta Delta C(T)) Method. *Methods* **25**, 402-408 (2001).

2 Roth, H. E., Bhinderwala, F., Franco, R., Zhou, Y. & Powers, R. DNAJA1 Dysregulates Metabolism Promoting an Antiapoptotic Phenotype in Pancreatic Ductal Adenocarcinoma. *J Proteome Res* **20**, 3925-3939 (2021).

3 Bolte, S. & Cordelieres, F. P. A guided tour into subcellular colocalization analysis in light microscopy. *J Microsc* **224**, 213-232 (2006).

4 Wojtkiewicz, M., Subramanian, S. P. & Gundry, R. L. Multinozzle Emitter for Improved Negative Mode Analysis of Reduced Native N-Glycans by Microflow Porous Graphitized Carbon Liquid Chromatography Mass Spectrometry. *Anal Chem* **96**, 5746-5751 (2024).

5 Doyle, C. P., Rectenwald, A., Timple, L. & Hammond, G. R. V. Orthogonal Targeting of SAC1 to Mitochondria Implicates ORP2 as a Major Player in PM PI4P Turnover. *Contact (Thousand Oaks)* **7**, 25152564241229272 (2024).

6 Li, J., Gui, Q., Liang, F. X. *et al.* The REEP5/TRAM1 complex binds SARS-CoV-2 NSP3 and promotes virus replication. *J Virol* **97**, e0050723 (2023).
